# Predominant contribution of *cis-*regulatory divergence in the evolution of mouse alternative splicing

**DOI:** 10.15252/msb.20145970

**Published:** 2015-07-01

**Authors:** Qingsong Gao, Wei Sun, Marlies Ballegeer, Claude Libert, Wei Chen

**Affiliations:** 1 Laboratory for Systems Biology and Functional Genomics, Berlin Institute for Medical Systems Biology, Max-Delbrück-Centrum für Molekulare MedizinBerlin, Germany; 2 Inflammation Research Center, VIBGhent, Belgium; 3 Department of Biomedical Molecular Biology, University GhentGhent, Belgium

**Keywords:** alternative splicing, *cis*-regulation, evolution

## Abstract

Divergence of alternative splicing represents one of the major driving forces to shape phenotypic diversity during evolution. However, the extent to which these divergences could be explained by the evolving *cis*-regulatory versus *trans*-acting factors remains unresolved. To globally investigate the relative contributions of the two factors for the first time in mammals, we measured splicing difference between C57BL/6J and SPRET/EiJ mouse strains and allele-specific splicing pattern in their F1 hybrid. Out of 11,818 alternative splicing events expressed in the cultured fibroblast cells, we identified 796 with significant difference between the parental strains. After integrating allele-specific data from F1 hybrid, we demonstrated that these events could be predominately attributed to *cis*-regulatory variants, including those residing at and beyond canonical splicing sites. Contrary to previous observations in *Drosophila*, such predominant contribution was consistently observed across different types of alternative splicing. Further analysis of liver tissues from the same mouse strains and reanalysis of published datasets on other strains showed similar trends, implying in general the predominant contribution of *cis*-regulatory changes in the evolution of mouse alternative splicing.

## Introduction

Alternative splicing (AS) generates multiple transcripts from the same gene by different combinations of exons, thereby increasing transcriptome plasticity and proteome diversity (Nilsen & Graveley, 2010). Recent studies using high-throughput sequencing indicate that about 25, 60 and 90% of multi-exon genes in *Caenorhabditis elegans*, *Drosophila melanogaster* and humans, respectively, undergo AS (Pan *et al*, 2008; Wang *et al*, 2008; Gerstein *et al*, 2010; Graveley *et al*, 2011; Ramani *et al*, 2011). Often in tissue and developmental stage-specific manner, AS is regulated by the interaction between *trans*-acting RNA binding proteins (RBPs) and *cis*-regulatory elements within nascent transcripts, including the well-defined 5′/3′ splice sites and branch sites as well as more diversified exonic/intronic splicing enhancers/silencers (Wang & Burge, 2008; Chen & Manley, 2009; Kalsotra & Cooper, 2011; Fu & Ares, 2014; Jangi & Sharp, 2014).

Changes in AS represent one of the major driving forces underlying the evolution of phenotypic differences across different species (Keren *et al*, 2010; Barbosa-Morais *et al*, 2012; Merkin *et al*, 2012; Lappalainen *et al*, 2013; Necsulea & Kaessmann, 2014). Such changes could arise from the divergences in *cis*-regulatory elements and/or *trans*-acting RBPs. The divergences of the two factors with different extent of pleiotropic consequences undergo distinct evolutionary trajectories. Therefore, to better understand evolution in AS, it is important to distinguish the relative contributions of *cis*- and *trans*-effects.

Several studies have tried to address this question in different species. However, it remains under debate which factor plays more important role in the evolution of AS, including skipped exons (SE), retained introns (RI), mutually exclusive exons (MXE), alternative 5′ splice sites (A5SS) and alternative 3′ splice sites (A3SS). Li *et al* studied genetic variation of AS in *Caenorhabditis elegans* by comprehensively identifying quantitative trait loci affecting the differential expression of transcript isoforms in a large recombinant inbred population. In total, they found only 22 genes showing evidence for genetic variation of AS, 77% of which were locally regulated, indicating a predominant contribution of *cis*-effects (Li *et al*, 2010). A more recent study in *Drosophila* used RNA-seq to investigate splicing regulatory evolution among species and showed that whereas RI, A3SS, and A5SS were primarily *cis*-directed, *trans*-effect had greater impacts on SE (McManus *et al*, 2014). In mammals, early work by Lin *et al*, based on the observation of higher sequence divergence flanking divergent SE events, suggested that changes in *cis*-regulatory elements made the major contribution to splicing divergence between human and chimpanzees (Lin *et al*, 2010). In the study by Barbosa Morais *et al*, the investigation of the splicing pattern of 13 human genes in a mouse strain carrying the majority of human chromosome 21 indicated that *cis*-regulatory changes were sufficient to drive the majority of species-specific pattern of exon inclusion/exclusion between human and mouse (Barbosa-Morais *et al*, 2012). Although these two mammalian studies implicated a predominant role of *cis*-divergence in the evolution of divergent exon-skipping events, a direct measurement of global contributions of *cis*- and *trans*-effects toward divergence of AS in mammals is still lacking. Particularly given the different *cis*-/*trans*- contributions to different types of AS observed in *Drosophila,* it remains unclear whether the same holds true in mammals.

To globally investigate the relative contribution of *cis*- and *trans*-regulatory changes for the first time in a mammalian system, we used RNA-seq to study splicing difference between *Mus musculus* C57BL/6J and *Mus spretus* SPRET/EiJ inbred mouse strains, as well as the allele-specific splicing pattern in their F1 hybrid. In F1 hybrids, the nascent RNA transcripts from both parental alleles are subject to the same *trans*-regulatory environments; thus, observed differences in allele-specific splicing pattern should only reflect the impact of *cis*-regulatory divergence. The contribution of *trans*-regulatory elements can then be inferred by comparing the allele-specific differences with the total splicing differences between the parental strains (Wittkopp *et al*, 2004, 2008; Springer & Stupar, 2007; Tirosh *et al*, 2009; Emerson *et al*, 2010; McManus *et al*, 2010, 2014; Goncalves *et al*, 2012; Coolon *et al*, 2014). The two parental strains chosen in this study diverged ∼1.5 million years (Ma) ago, which resulted in about 35.4 million single nucleotide variants (SNVs) and 4.5 million insertion and deletions (indels) between their genome sequences (Dejager *et al*, 2009; Keane *et al*, 2011). Such a high sequence divergence allows us to unambiguously determine the allelic origin for a large fraction of short RNA-seq reads, thereby enables accurate quantification of allelic pattern for thousands of splicing events. In total, we identified 796 (6.7%) differentially regulated splicing events between the two parental strains. By comparing them to allele-specific splicing pattern in F1 hybrid, we could attribute such splicing divergency predominately to *cis*-regulatory variants, including those residing at and beyond canonical splicing sites. In contrast to the observation in *Drosophila*, such predominant contributions of *cis*-regulatory changes were consistently observed across different types of AS. Further analysis of liver tissues in the same parental and F1 hybrid strains showed a same trend. Importantly, reanalysis of published RNA-seq datasets generated from the livers of C57BL/6J, CAST/EiJ, and their F1 hybrids demonstrated again predominant contributions of *cis*-regulatory changes for all five AS types, implying such conclusion could be generalized to the evolution of AS in mouse.

## Results

### Divergence in alternative splicing between C57BL/6J and SPRET/EiJ

**Figure 1:**
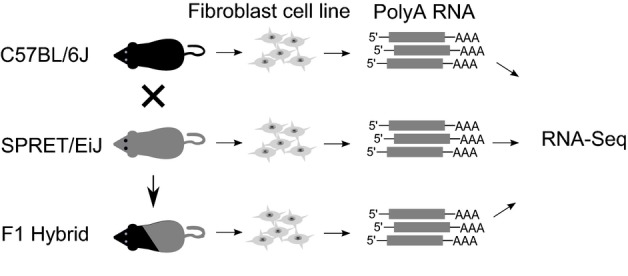
Study design Fibroblast cells were isolated from adult C57BL/6J, SPRET/EiJ, and the F1 hybrid mice and cultured. PolyA RNAs prepared from each cell line were sequenced on an Illumina HiSeq 2000/2500 platform.

To characterize the divergence of alternative splicing between C57BL/6J and SPRET/EiJ, we derived fibroblast cell lines from the two mouse strains and sequenced three biological replicates of polyA RNAs isolated from them on an Illumina HiSeq 2000/2500 platform (Fig1, Materials and Methods). Paired-end sequencing resulted in an average of 169.4 million read pairs from each parental sample (Table EV1). These reads were then mapped to the corresponding genome using a splicing-aware alignment tool TopHat (Materials and Methods) (Trapnell *et al*, 2009).

After mapping, a previously developed Bayesian inference methodology—Mixture of Isoforms (MISO)—was applied for quantification (measured by Percent Spliced In, PSI) and comparison (ΔPSI) of alternative splicing events between C57BL/6J and SPRET/EiJ (Katz *et al*, 2010). Five major types of alternative splicing events were considered: SE, RI, MXE, A5SS, and A3SS. A total of 30,199 annotated splicing events in mouse genome downloaded from MISO Web page (http://genes.mit.edu/burgelab/miso) were considered in this study (Table EV2). To ensure higher accuracy, we required the quantification of a splicing event to be supported with at least 20 sequencing reads in all samples. In total, 11,818 events were retained for further analysis, including 5,615 SE, 1,768 RI, 696 MXE, 2,236 A3SS, and 1,503 A5SS (Table EV2, Materials and Methods).

We utilized the Bayesian factor (BF) as a measure of statistical significance for splicing difference (ΔPSI). After applying a threshold of BF > 5 in all the three replicates and average |ΔPSI| > 0.1, a criterion previously shown to maximize the number of significant events and minimize the false discovery rate (Sterne-Weiler *et al*, 2013), we identified in total 796 events showed significant splicing divergence between the two parental strains (Table1 and FigEV1, false discovery rate (FDR) = 2.5%). These divergent events covered all the five AS types (Table1).

**Table 1:** Comparison of alternative splicing between C57BL/6J and SPRET/EiJ

	Total expressed events	Differential events (%)	*P*-value (Fisher’s exact test)
Total number	11,818	796 (6.7)	
Event type			
SE	5,615	418 (7.4)	
RI	1,768	124 (7.0)	
A3SS	2,236	101 (4.5)	
A5SS	1,503	99 (6.6)	
MXE	696	54 (7.8)	
Event effect			
Non-coding regions^a^	3,400	317 (9.3)	1.1e-10
Coding regions	8,418	479 (5.7)	
Frame-neutral events	4,235	273 (6.4)	4.8e-3
Frame-shifting events	4,183	206 (4.9)	

a Non-coding regions include non-coding genes and untranslated regions (UTRs) of coding genes.

**Figure EV1:**
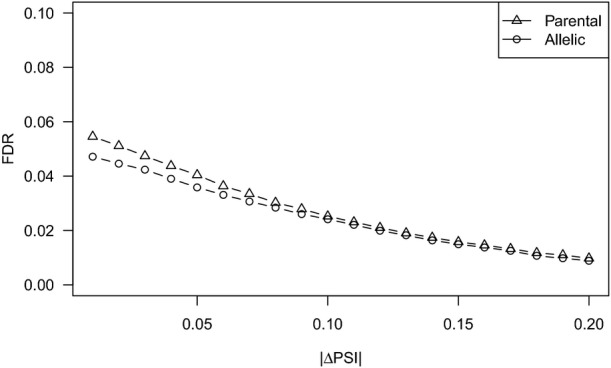
FDR estimation for each |ΔPSI| cutoff FDR for parental (triangle) and allelic (circle) splicing comparison (*y*-axis) was plotted against different |ΔPSI| cutoffs (*x*-axis). For each value of *x* from 0.01 to 0.20 increasing by 0.01, we performed independent 100 bootstrapped label permutations of replicate 2 and replicate 3, respectively. For each of the 100 shuffled sets, we calculated the number of events passing the threshold (false positives), *that is* BF > 5 in all the replicates and average |ΔPSI| > *x*. Then, for each of the 100 permutations of each value *x*, the FDR was estimated as false positives divided by the number of real events passing the threshold, including both false positives and true positives.

Alternative splicing can affect either protein-coding sequences or non-coding ones (including non-coding genes and untranslated regions of coding genes). The former might be subject to stronger selection during evolution. Consistent with this, among the divergent AS events, the frequency of divergent splicing in non-coding regions was significantly higher than that in coding region (Table1). Furthermore, within the set of divergent event in protein-coding regions, frame-preserving events were more likely to be divergent compared to frame-shifting events. These results demonstrated that in general AS with functional relevance was under stronger negative selection.

### Predominant contribution of *cis*-regulatory variants underlying divergent AS between C57BL/6J and SPRET/EiJ

Alternative splicing divergence between species can arise from *cis*- and/or *trans*-regulatory differences. After identifying alternative splicing differences between the two parental strains, we next addressed the relative contributions of *cis*-regulatory differences in AS divergence using their F1 hybrids. *Trans*-acting contributions can then be inferred by comparing allele-specific differences in the hybrid to the splicing differences between the parental strains.

Paired-end sequencing of polyA RNAs isolated from F1 fibroblast cell line resulted in on average 388.0 million read pairs for each of the three replicates (Table EV1). The high density of sequence variants between the genomes of C57BL/6J and SPRET/EiJ allowed the unambiguous assignment of allelic origin for an average of 180.6 million read pairs in each replicate, which were used for further quantification of allelic alternative splicing (Table EV1).

To avoid bias due to the potential misalignment of reads to the wrong allele, we first created a mock F1 hybrid RNA-seq dataset by mixing equal amounts of RNA-seq reads derived from the two parental strains. We then compared the PSI values of 11,818 expressed splicing events for both strains estimated based on the separate RNA-seq data from the parental strains to the allelic PSI values calculated using only those reads in the mock F1 dataset that could be unambiguously assigned to either allele. A total of 2,595 events supported with < 20 allelic reads in the mock dataset and 2,689 events with significant difference between the two PSI values for either allele were filtered out (FigEV2A and B, Materials and Methods). FigureEV2C–E shows that for the remaining 6,534 “well-behaved” events, both the PSI and ΔPSI values in the parental strains correlated well with the allele-specific values in mock F1 hybrid.

**Figure EV2:**
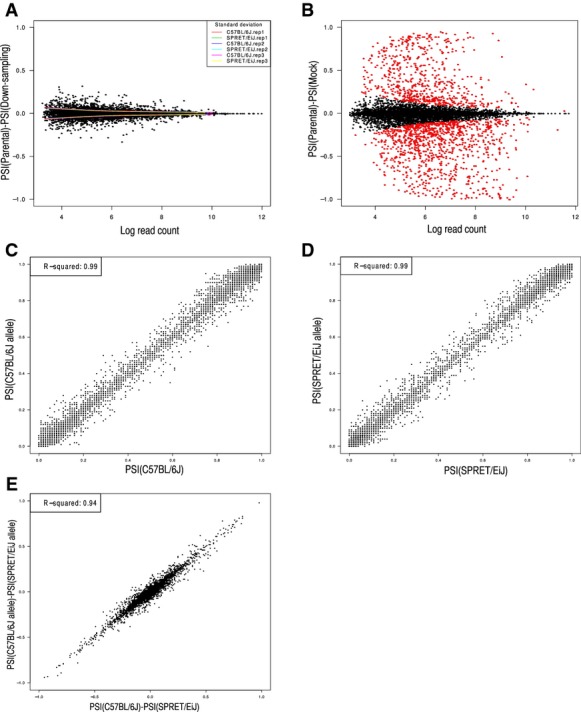
Illustration of data filtering based on mock F1 hybrid A MA plot comparing the PSI values in parental strains and their downsampling datasets. The local standard deviation for each comparison was also indicated (see Materials and Methods).

B MA plot comparing the PSI values in parental strains and those estimated based on mock F1 dataset. The red dots represented the outliers with inconsistent PSI values between parental strain and mock F1 dataset.

C–E After filtering, the PSI values for C57BL/6J (C), SPRET/EiJ (D), and their difference (E) correlated well between parental strains and mock F1 hybrid (*R*^2^ = 0.99, 0.99 and 0.94, respectively).

**Figure 2:**
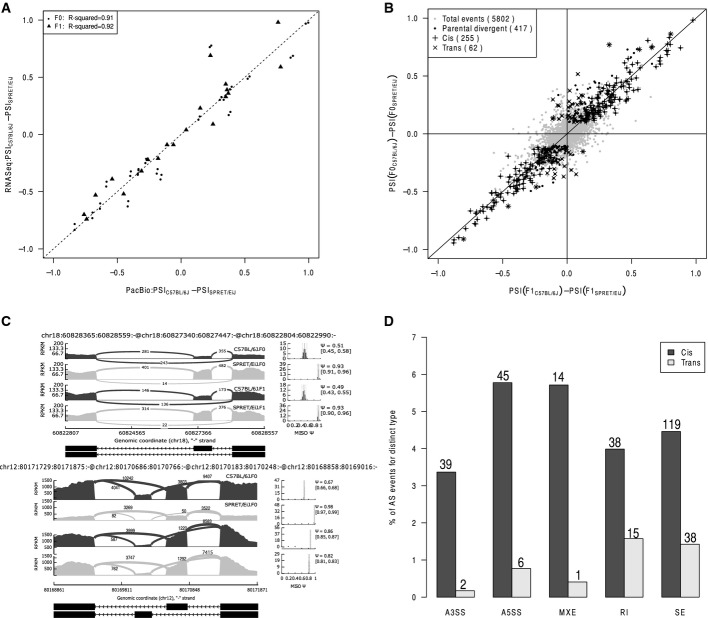
Dissection of *cis*- and *trans*-regulatory contributions in alternative splicing Scatterplot comparing parental splicing differences (dots, denoted as F0 hereafter) or allelic splicing differences (triangles) estimated based on Illumina RNA-seq results (*y*-axis) to those based on PacBio sequencing of splicing event-spanning cDNA products (*x*-axis) (*R*^2^ = 0.91 and 0.92 for comparison of parental and allelic difference, respectively).

Out of 6,534 AS events, 5,802 supported with at least 20 sequencing reads in all three F1 hybrid sequencing replicates were retained for further analysis (Table EV2). After applying the same threshold as that for parental strain, *that is* BF > 5 in all the three replicates and average |ΔPSI| > 0.1, we could detect a total of 381 divergent events between the two alleles in F1 hybrid (FigEV1, FDR = 2.4%). To assess the accuracy of our allele-specific splicing analysis, we selected 20 candidate events consisting of all five different AS types (eight SE, three RI, three MXE, two A3SS, and four A5SS) for validation. Using PacBio RS system, we deep-sequenced the AS-spanning RT–PCR products amplified from either parental strains or F1 hybrid using primers targeted at flanking constitutive regions with no sequence variant between the two strains (FigEV3, Materials and Methods) (Eid *et al*, 2009; Sun *et al*, 2013). The full-length sequences could be used to assign the PacBio reads to different isoforms from different strain/alleles, which were then counted to calculate the strain/allele-specific PSI (Table EV3). As shown in Fig2A, the splicing changes estimated in this way were significantly correlated with those determined by RNA-seq (*R*^2^ = 0.92).

**Figure EV3:**
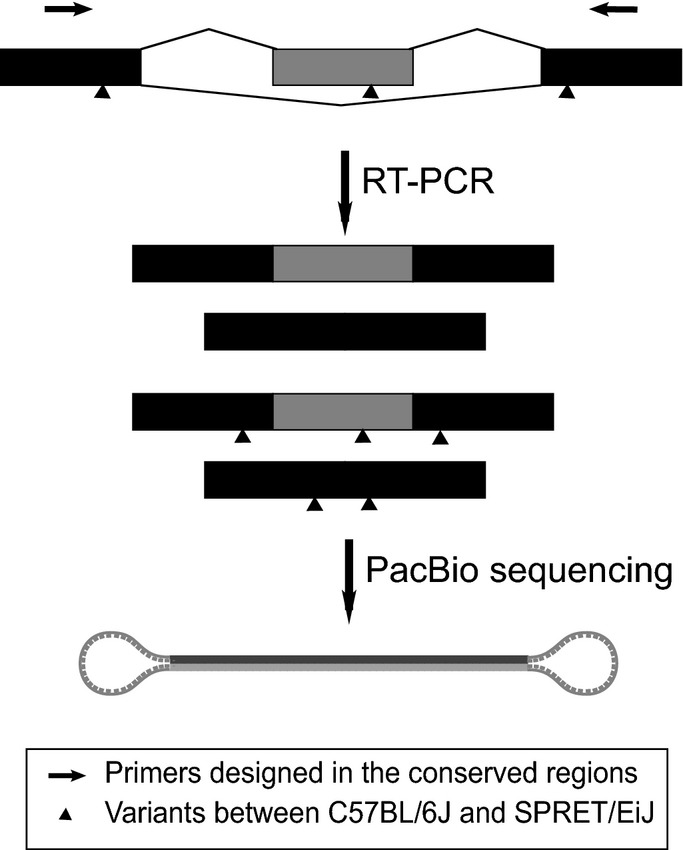
Illustration of PacBio sequencing of splicing event-spanning cDNA products For each candidate event, RT–PCR primers were designed in the conserved regions of the constitutive exons to amplify both isoforms from the two alleles/strains. The PCR products were then sequenced at full length using PacBio RS system.

Scatterplot comparing splicing difference in parental strains (*y*-axis) versus the allelic difference in F1 hybrid (*x*-axis). After filtering using mock F1 hybrid, 5,802 AS events were expressed in F1 hybrid (gray dots). Among these, 417 AS events were divergent between parental strains (black dots), of which 255 (indicated as “+”) and 62 (indicated as “×”) exhibited significant *cis*- and *trans*-regulatory divergence, respectively.

Examples of *cis* (upper panel)- and *trans* (lower panel)-regulatory divergence in alternative splicing. The RNA-seq read densities supporting the inclusion and exclusion of exons were shown in the left plot. The estimated PSI values and 95% confidence intervals were shown in the right plot.

Percentage of *cis-* and *trans*-divergent events for the five AS types separately (numbers of events for each type were indicated above bars).

We then compared the allelic divergent AS to the divergent AS between the parental strains. Out of 5,802 retained events, 417 had divergent regulation between parental strains, of which 255 and 62 exhibited *cis*- and *trans*-divergences, respectively (Fig2B, Materials and Methods). Figure2C shows two representative examples for the divergent splicing events with predominant *cis*- and *trans-*contributions, respectively. Such predominant *cis*-contributions were evident for all the five different types of AS (Fig2D).

To check whether our conclusion was sensitive to difference thresholds, we tried different cutoffs of |ΔPSI| values to determine the divergent AS events (FigEV1). As shown in FigEV4A–C, *cis*-regulatory divergence always showed predominant contribution at different thresholds (|ΔPSI|> 0.0, 0.05, and 0.15, respectively) and this trend also held true for all the five AS types (FigEV4D–F). Furthermore, we also checked whether the contributions of *cis-*/*trans*-regulatory divergence were different for parental divergent events with different effect sizes (|ΔPSI|). For this, we grouped the 417 divergent events between the parental strains into seven categories according to the |ΔPSI| values: (0.1, 0.2], (0.2, 0.3], (0.3, 0.4], (0.4, 0.5], (0.5, 0.6], (0.6, 0.7], and (0.7, 1.0]. As shown in FigEV4G, while *cis*-regulatory divergence always played the predominant role in determining parental AS divergence with different effect sizes, its relative contribution slightly decreased with the decreasing effect size.

**Figure EV4:**
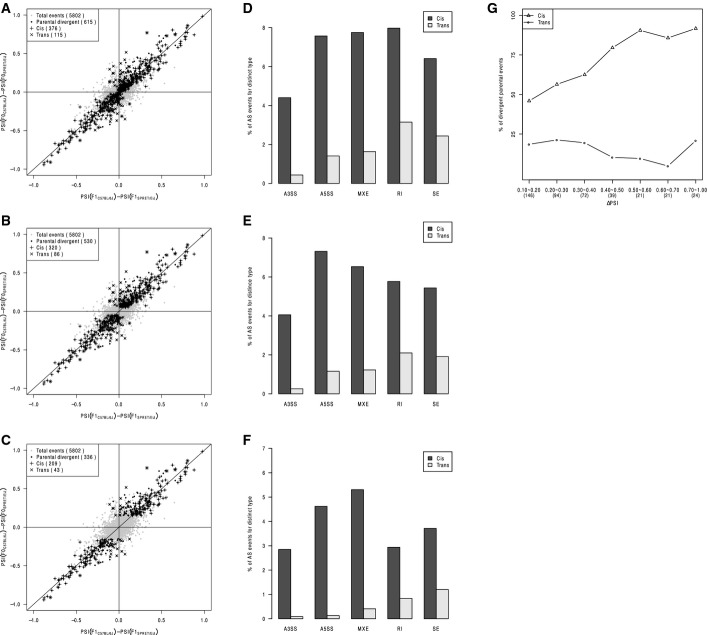
Dissection of *cis*- and *trans*-regulatory contributions in alternative splicing at different |ΔPSI| cutoffs A–C Scatterplot comparing splicing differences in parental strains (*y*-axis) versus the allelic differences in F1 hybrid (*x*-axis) at different |ΔPSI| cutoffs [|ΔPSI| > 0 (A), 0.05 (B) and 0.15 (C)]. After filtering using mock F1 hybrid, 5,802 AS events were expressed in F1 hybrid (gray dots). Among these, 615 (A)/530 (B)/336 (C) AS events were divergent between parental strains (black dots), of which 376 (A)/320 (B)/209 (C) (indicated as “+”) and 115 (A)/86 (B)/43 (C) (indicated as “×”) exhibited significant *cis*- and *trans*-regulatory divergence, respectively.

D–F Percentage of *cis-* and *trans*-divergent events for the five AS types separately at different |ΔPSI| cutoffs [|ΔPSI| > 0 (D), 0.05 (E), and 0.15 (F)].

G Contributions of *cis* (indicated as triangle)-/*trans* (indicated as circle)-regulatory divergence (*y*-axis) to parental divergent AS events with different effect sizes (|ΔPSI|, *x*-axis). A total of 417 divergent events between parental strains (see Fig2B) were grouped into 7 categories according to the |ΔPSI| values: (0.1, 0.2), (0.2, 0.3), (0.3, 0.4), (0.4, 0.5), (0.5, 0.6), (0.6, 0.7), and (0.7, 1.0). The number of events in each category was marked. While *cis*-regulatory divergence always played the predominant role in determining parental AS divergence with different effect sizes, its relative contribution slightly decreased with the decreasing effect size.

To check whether our conclusion could be affected by the specific statistical methods applied in this study, we tried a different statistical test—Fisher’s exact test—to determine the statistical significance in calculating splicing divergence. As shown in FigEV5A and B, more divergent events in both parental and allelic comparisons could be identified using Fisher’s exact test, and indeed, nearly all the significantly divergent events found by MISO could also be detected using Fisher’s exact test. We then compared the divergent AS identified by Fisher’s exact test in parental strains to those in F1 hybrid. As shown in FigEV5C and D, *cis*-regulation showed again predominant contributions for all the five AS types, demonstrating that our conclusion on predominant *cis-*contribution in splicing divergence was not test-dependent.

**Figure EV5:**
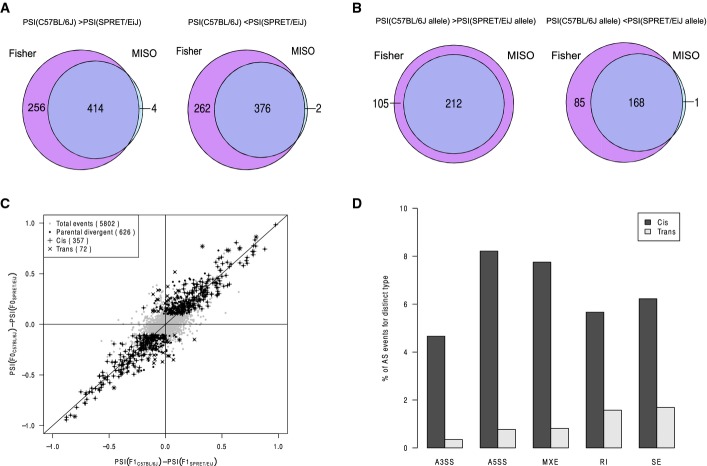
Divergent AS events identified using Fisher’s exact test A, B Venn diagram showing the overlap of the divergent events identified by Fisher’s exact test and MISO in parental strains (A) and in F1 hybrid (B).

C Scatterplot comparing splicing difference in parental strains versus the allelic difference in F1 hybrid identified by Fisher’s exact test. After filtering using mock F1 hybrid, 5,802 AS events were expressed in F1 hybrid (gray dots). Among these, 626 AS events were divergent between parental strains (black dots), of which 357 (indicated as “+”) and 72 (indicated as “×”) exhibited significant *cis*- and *trans*-regulatory divergence, respectively.

D Percentage of *cis*- and *trans*-divergent events for the five AS types separately using Fisher’s exact test.

To check whether our conclusion from cultured cells could be extended to mouse tissues, we performed RNA-seq on two replicates of the liver samples from C57BL/6J, SPRET/EiJ, and their F1 hybrid, respectively (Table EV1). Out of 8,759 AS events expressed in the parental samples, 607 were identified as significantly divergent between the parental strains (BF > 5 in both replicates and average |ΔPSI|> 0.1). After the similar filtering based on mock F1 dataset, 4,124 and 336 total expressed and divergent events retained, respectively (Table EV2). Then by applying the same threshold as that for parental strains, we detected 270 divergent events between the two alleles in F1 hybrid (Table EV2). Finally, we compared the allelic divergent to the parental divergent AS. Out of 336 parental divergent events retained after filtering, 196 and 38 exhibited significant *cis*- and *trans*-regulatory divergence, respectively (FigEV6A). Such predominant contributions of *cis*-regulatory divergence were also evident for all the five splicing types (FigEV6B).

**Figure EV6:**
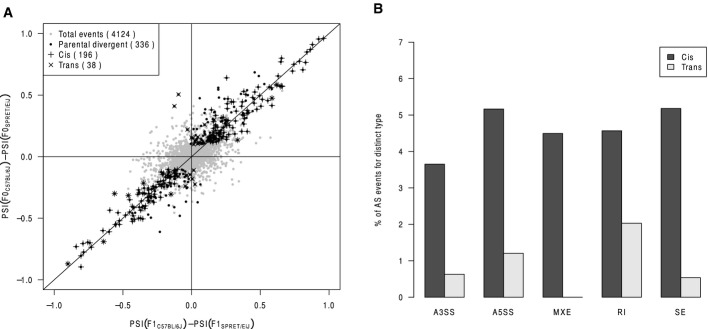
Dissection of *cis*- and *trans*-regulation in alternative splicing between C57BL/6J and SPRET/EiJ liver samples Scatterplot comparing splicing difference between C57BL/6J and SPRET/EiJ liver samples versus their allelic difference in F1 hybrid liver sample. After filtering using mock F1 hybrid, 4,124 AS events were expressed in F1 hybrid (gray dots). Among these, 336 AS events were divergent between parental strains (black dots), of which 196 (indicated as “+”) and 38 (indicated as “×”) exhibited significant *cis*- and *trans*-regulatory divergence, respectively.

Percentage of *cis*- and *trans*-divergent events for the five AS types separately.

To check whether our conclusion could be generalized to other mouse strains, we compared the AS patterns between C57BL/6J and CAST/EiJ using previously published dataset (Goncalves *et al*, 2012). These two strains diverged about 1 Ma ago, resulting in 17.7 million SNVs and 2.7 million indels between their genome sequences (Keane *et al*, 2011). The lower density of sequence variants, together with shorter sequencing reads (2 × 72nt), allowed in their F1 hybrid RNA-seq data only about 30.2% of the mappable reads to be unambiguously assigned to their parental alleles (compared to about 61.1% in our F1 hybrid of C57BL/6J and SPRET/EiJ, Table EV1). Therefore, to obtain a sufficient number of reads for accurate PSI quantification, we pooled the data from three individuals together and generated two replicate datasets for C57BL/6J, CAST/EiJ, and their F1 hybrid, respectively (Materials and Methods). We then performed the same analysis as described before. Although the absolute numbers of divergent events identified both between parental strains and between alleles in F1 hybrid were understandably lower, the predominant contribution of *cis*-regulatory divergence (44 *cis* versus six *trans*) was still evident (FigEV7A), and this trend held true for all the five splicing types (FigEV7B). This implied, in general, predominant *cis*-contribution in the evolution of mouse alternative splicing.

**Figure EV7:**
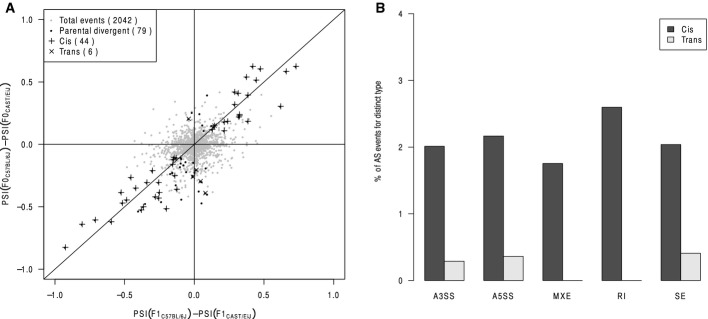
Dissection of *cis*- and *trans*-regulation in alternative splicing between C57BL/6J and CAST/EiJ Scatterplot comparing splicing difference between C57BL/6J and CAST/EiJ versus their allelic difference in F1 hybrid. After filtering using mock F1 hybrid, 2,042 AS events were expressed in F1 hybrid (gray dots). Among these, 79 AS events were divergent between parental strains (black dots), of which 44 (indicated as “+”) and 6 (indicated as “×”) exhibited significant *cis*- and *trans*-regulatory divergence, respectively.

Percentage of *cis*- and *trans*-divergent events for the five AS types separately.

### Genomic features that correlate with *cis*-regulatory AS divergence

**Figure 3:**
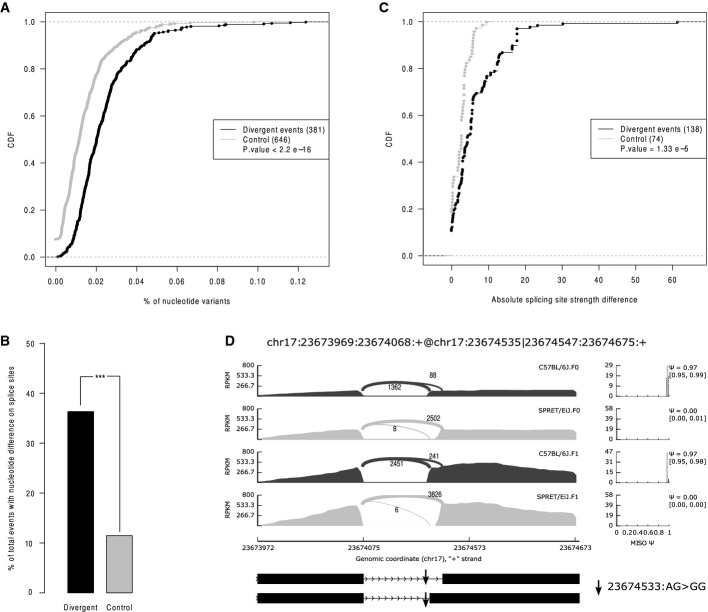
Genomic features that correlate with *cis*-regulatory alternative splicing divergence The cumulative distribution function (CDF) of frequencies of nucleotide variants in the AS flanking regions for the events with *cis*-regulatory divergence (black) and controls (grey). Compared with controls, the events with significant *cis*-regulatory impact had higher sequence divergence in the flanking regions. The *P*-values were calculated by the Mann–Whitney *U*-test.

*Cis*-regulatory divergence should result solely from sequence variants in pre-mRNA sequences, particularly those residing close to the affected splicing events. To investigate this, we calculated the frequencies of SNVs and indels in the regions flanking the AS events with or without *cis*-regulatory divergence (FigEV8). As shown in Fig3A, compared with those without *cis*-divergence (control events, see Materials and Methods), the regions flanking AS events with *cis*-divergence contained significantly higher density of sequence variants between the two strains (see also Fig EV9A for the comparison of different AS types separately).

**Figure EV8:**
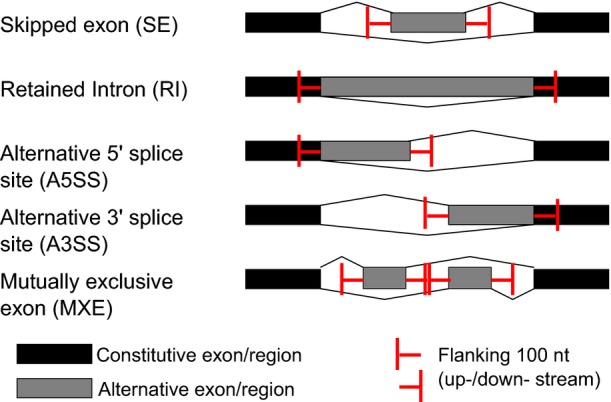
Illustration of the regions flanking the AS events For SE, the alternative exons and their flanking 100 nt intron sequences were considered; for RI, the retained introns and their flanking 100 nt exon sequences were considered. For A3SS or A5SS, the alternative exon regions and their flanking 100 nt exon/intron sequences were considered. For MXE, both alternative exons and their flanking 100 nt intron sequences were considered.

36.2 and 11.5% of the events with significant *cis*-regulatory divergence (black) and control events (gray) had sequence divergence at their exact splice sites, respectively (***P = 9.21e-14, Fisher’s exact test).

CDF of allelic differences in splicing site strengths due to sequence variants at the exact splicing sites plotted for *cis*-regulatory divergent events (black) and control events (grey), separately. The splicing site strengths changed more in the events with *cis*-regulatory events than in those without. The *P*-values were calculated by the Mann–Whitney *U*-test.

An example showing that a SNV at the canonical GU/AG sites (indicated as an arrow) resulted in complete functional abortion of the corresponding splice sites. The substitution of the AG to GG in SPRET/EiJ disrupted the splicing site and thereby facilitated the use of a downstream splicing acceptor.

**Figure EV9:**
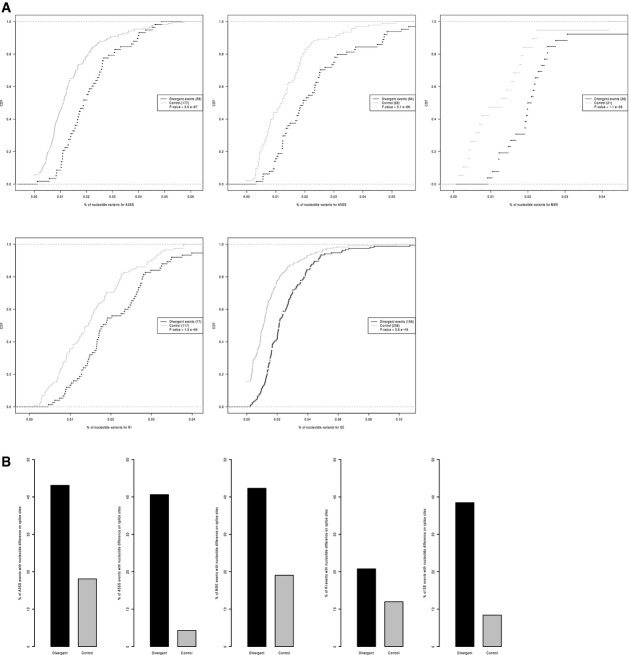
Genomic features that correlate with *cis*-regulatory alternative splicing divergence for each AS type separately CDF of frequencies of nucleotide variants in the AS flanking regions for the events with *cis*-regulatory divergence (black) and controls (grey) for A3SS, A5SS, MXE, RI, and SE, respectively.

Percentages of the events with significant *cis*-regulatory divergence (black) and controls events (gray) that had sequence divergence at the exact splice sites for A3SS, A5SS, MXE, RI, and SE, respectively.

**Figure EV10:**
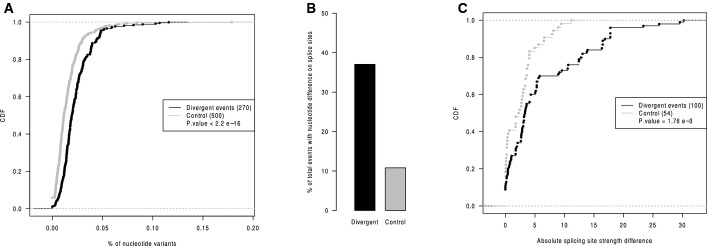
Genomic features that correlate with *cis*-regulatory alternative splicing divergence identified in the liver sample CDF of frequencies of nucleotide variants in the AS flanking regions for the events with *cis*-regulatory divergence (black) and controls (grey) identified in liver sample. Compared with controls, the events with significant *cis*-regulatory impact also had higher sequence divergence in the flanking regions.

We then checked how sequence variants at the exact splicing sites could contribute to the events with *cis*-regulatory divergence. As shown in Fig3B, 36.2% of these events with *cis*-regulatory divergence had at least one sequence variants at the respective splicing sites, compared to 11.5% of control events (*P* = 9.2e-14, Fisher’s exact test, see also Fig EV9B for the comparison of different AS types separately). Sequence variants at splice sites could regulate alternative splicing by affecting splice site strength—the probability that the splice sites could be recognized by the spliceosome (McManus *et al*, 2014). To investigate how sequence variants at the splicing sites could affect splicing site strength, we calculated the splicing site strength score for the two alleles containing variants at the exact splice sites (Materials and Methods) and compared the allelic difference of such score between the events with *cis*-regulatory divergence and those without. As shown in Fig3C, the sequence variants at the splicing sites of *cis*-divergent events affected the splicing site strength more than those at splicing sites of control events. As expected, variants changing the canonical GU/AG splicing donor/acceptor sites severely affected the splicing site strength, which resulted in complete functional abortion of the corresponding splicing site, as exemplified in Fig3D. Importantly, the same analysis of the liver data showed a similar correlation of all these genomic features (Fig EV10). Taken together, sequence variants at the canonical splicing sites could affect splicing site strength and thereby lead to divergent AS.

In liver sample, 37.0 and 10.8% of the events with significant *cis*-regulatory divergence (black) and controls (gray) had sequence divergence at the exact splice sites, respectively.

CDF of allelic differences in splicing site strengths due to sequence variants at the exact splicing sites plotted for *cis*-regulatory divergent events (black) and controls (grey) identified in liver sample. The splicing site strengths changed more in the events with *cis*-regulatory events than in those without.

**Figure 4:**
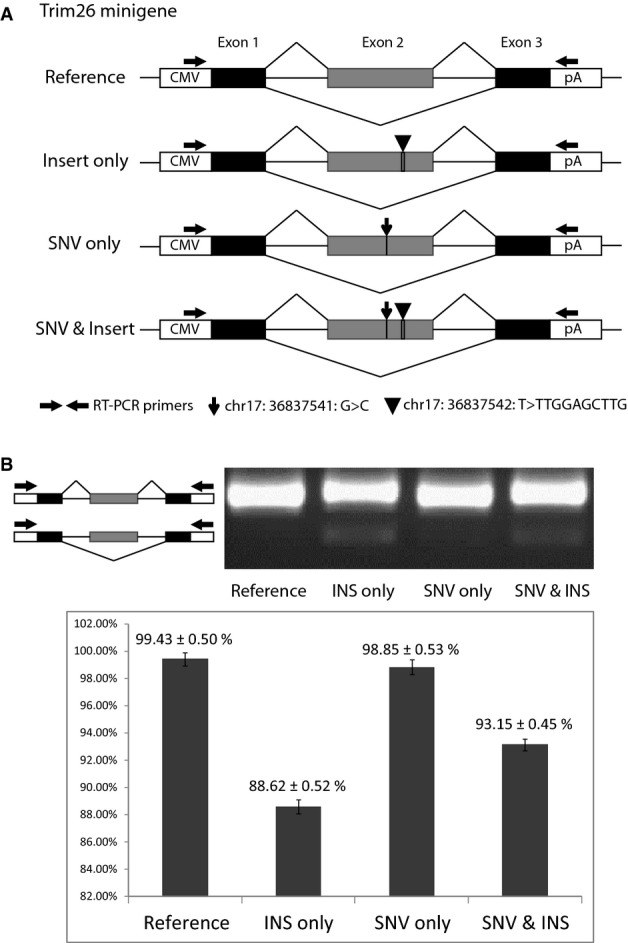
Minigene analysis for the *cis*-divergent SE event in Trim26 gene Schematic diagrams of minigene constructs for validating the *cis*-divergent SE event identified in Trim26 gene. Two candidate variants, one SNV and one insertion (INS), were indicated. Four constructs were prepared in C57BL/6J background with no variant, only insertion, only SNV, and both insertion and SNV, respectively (see Materials and Methods).

*Cis*-regulatory variants could affect as well the regulatory elements beyond canonical splicing sites, such as exonic/intronic splicing enhancers/silencers. To identify the regulatory elements underlying these *cis*-divergent AS that we observed, we focused on those 243 *cis*-divergent events without sequence variants at the splicing sites (Table EV4). On average, about 12 variants were found within the exon/intron regions flanking each of these events. To determine the exact functional variant(s), we integrated published RNA-seq datasets from brain tissue of five mouse strains (C57BL/6NJ, CAST/EiJ, PWK/PhJ, WSB/EiJ, and SPRET/EiJ) (Danecek *et al*, 2012). Five events showed consistent splicing patterns between brain tissues and fibroblast cell line for both C57BL/6J and SPRET/EiJ strains (|ΔPSI| ≤ 0.1, Table EV4). By correlating the sequence variants with splicing patterns across different mouse strains, we could identify a total of 11 candidate variants potentially responsible for these events (see Table EV4 for details). To confirm the relevance of our finding, we chose one divergent SE in Trim26 gene for further analysis. As shown in FigEV11, there were in total four sequence variants in the regions flanking the divergent SE, two of which followed the splicing pattern across different mouse strains, including one 9-nucleotide (nt) insertion and one SNV (Table EV4 and FigEV11). To assess which of the two variants contributed to the divergent splicing pattern, we investigated their effects using minigene reporter assays. Four different minigene constructs containing different combinations of these two variants were transfected into Hek293T and 3T3 cells: (i) “reference”: containing no variant compared to C57BL/6J genome; (ii) “insert only”: containing only the SPRET/EiJ insertion variant; (iii) “SNV only”: containing only the SPRET/EiJ SNV variant; (iv) “SNV & insert”: containing both the SPRET/EiJ insertion and SNV variants (Fig4A, Materials and Methods). As shown in Fig4B and Fig EV12, the splicing differences detected between “reference” and “SNV & insert” constructs were consistent with the splicing divergence observed between C57BL/6J and SPRET/EiJ strains; *that is,* the PSI values from SPRET/EiJ allele were smaller than those from the C57BL/6J allele. Further comparison of “insert only” and “SNV only” constructs showed that the insertion variant alone could lead to the enhanced SE observed in SPRET/EiJ allele.

**Figure EV11:**
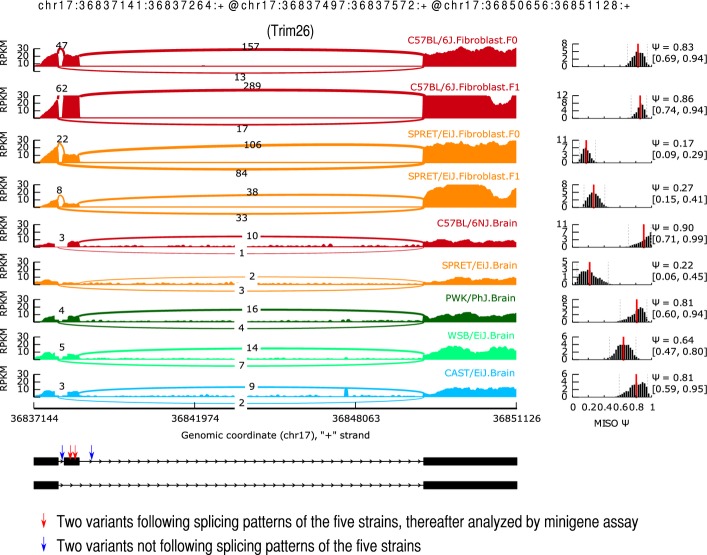
Sashimi plot for the splicing patterns of the SE event in Trim26 gene from fibroblast cell line as well as brain tissues of five mouse strains The top four rows represented splicing patterns for C57BL/6J and SPRET/EiJ strains and their alleles in F1 hybrid. The bottom five rows represented splicing patterns for brains tissues of the five mouse strains. PWK/PhJ and CAST/EiJ had a similar splicing pattern as C57BL/6J, but different from SPRET/EiJ. Four variants located in the flanking regions, two of which correlated with the species-specific splicing pattern and were analyzed using minigene assays (see Fig4).

Minigene assays of the four constructs transfected into HEK293T cells suggested only the insertion contributed to this divergent SE event. The gel image illustrated RT–PCR products from these constructs. The barplot below the gel image represented the PSI values calculated from triplicates of RT–PCR products using Agilent Bioanalyzer 2000 system (see Materials and Methods, for minigene assays in NIH3T3 cells, see FigEV12).

**Figure EV12:**
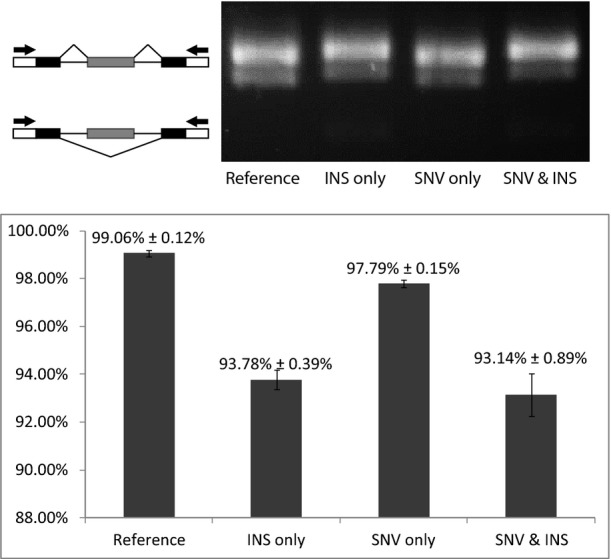
Minigene analysis for the *cis*-divergent SE event in Trim26 gene in NIH3T3 cells Label is the same as in Fig4B.

## Discussion

Change in AS, one of the major driving forces to shape phenotypic diversity during evolution, could arise from the divergences in *cis*-regulatory elements and/or *trans*-acting RBPs. To globally investigate the relative contributions of the two factors for the first time in a mammalian system, we applied RNA-seq to investigate splicing difference between C57BL/6J and SPRET/EiJ inbred mouse strains and allele-specific splicing pattern in their F1 hybrid. Our results clearly showed the predominant contribution of *cis*-regulatory variants across all the five types of AS.

To identify the genetic variants with regulatory effects on gene expression, the most popular method is expression quantitative trait loci (eQTL) mapping, in which different genotypes are correlated with gene expression level in a large population with diverse genetic backgrounds (Pickrell *et al*, 2010; Majewski & Pastinen, 2011; Lappalainen *et al*, 2013). Recently, this strategy has been extended to measure the genetic regulation on AS (asQTL) (Li *et al*, 2010). However, genome-wide eQTL/asQTL mapping that test the association between all SNPs against all expression/AS events are statistically underpowered, in particular for identifying *trans*-factors lying in distal regions. Therefore, the relative *cis*-/*trans*-contributions estimated using QTL methods could be biased toward higher *cis-*effects. An alternative approach that could more directly address the effect of *cis*-/*trans*-divergences is to compare the allelic difference in F1 hybrid to the difference observed between two parental strains. This approach has been successfully used for studying *cis-*/*trans-*contribution in gene expression divergence in yeast, fly, mouse, and plant (Wittkopp *et al*, 2004, 2008; Springer & Stupar, 2007; Tirosh *et al*, 2009; Emerson *et al*, 2010; McManus *et al*, 2010; Goncalves *et al*, 2012; Coolon *et al*, 2014). More recently, McManus *et al* used this strategy to address the *cis*-/*trans*-contribution to AS evolution in *Drosophila* (McManus *et al*, 2014). In this study, we applied the same approach in mice and chose C57BL/6J, SPRET/EiJ, and their F1 hybrid as our model. Among all the mouse strains with high-quality genome assembly, SPRET/EiJ has the largest number of sequence variants relative to C57BL/6J. Their sequence variants are about twice as many as those between CAST/EiJ and C57BL/6J, the two strains used in previous allele-specific gene expression analysis (Goncalves *et al*, 2012). This large genomic divergence first provides a large number of potential regulatory variants between the two strains. Second, more importantly, it allows the sequencing approach to distinguish allelic RNA transcripts. In our study, about 60% of mapped 2 × 100nt reads could be unambiguously assigned to their parental alleles. Moreover, the allelic ΔPSI value correlated well with independent measurement using PacBio full-length sequencing of AS-spanning cDNA products (*R*^2^ = 0.92).

In cultured fibroblast cells, we identified 796 and 381 differentially regulated splicing events between the two parental strains and between the two alleles in F1 hybrid, respectively. By comparing the two datasets, we could attribute the splicing divergence between the two strains predominately to *cis*-regulatory variants for all five types of AS. Importantly, a similar analysis on the liver tissues from the same parental and F1 strains showed a same trend. To further exclude the possibility that our observation of predominant *cis*-contribution was a peculiarity of the two mouse strains used in this study, we reanalyzed published RNA-seq datasets generated from the liver of C57BL/6J, CAST/EiJ, and their F1 hybrid (Goncalves *et al*, 2012). Although the absolute number of divergent events both between parental strains and between alleles in F1 hybrid that we could identify was much lower, the predominant contribution of *cis*-regulatory difference was still evident, implying the predominant *cis*-contribution could be generalized to the evolution of AS in mouse.

Our observation was consistent with previous study of difference in exon-skipping between human and mouse, in which 13 divergent SE events were mostly attributed to *cis*-regulatory variants (Barbosa-Morais *et al*, 2012). In contrast, a more recent study in *Drosophila* found that whereas RI, A3SS, and A5SS were still primarily *cis*-directed, *trans*-effects played a dominant role in SE divergence. The authors of latter study attributed the inconsistence between their result and the result from human/mouse study to the different evolutionary distances, *that is* ∼2.5 Ma between different *Drosophila* strains versus ∼75 Ma between human and mouse (Waterston *et al*, 2002; Cutter, 2008; McManus *et al*, 2014). *Cis*-regulatory divergences could preferentially accumulate over evolutionary time and therefore contribute more substantially to the human/mouse comparison (Lemos *et al*, 2008; Wittkopp *et al*, 2008). However, in our study, the evolutionary distance between C57BL/6J and SPRET/EiJ strains is ∼1.5 Ma, similar as that in the *Drosophila* study. Thus, our results of consistent *cis*-dominant contribution excluded different evolutionary distances as a plausible explanation for inconsistent observations between *Drosophila* and mammals. Instead, a more plausible explanation for the discrepancy is genuine differences in mechanisms underlying evolutions of AS regulations between *Drosophila* and mammals. Previous studies have demonstrated the splicing evolutions differ from several perspectives between *Drosophila* and mouse (Xiao *et al*, 2007; Khodor *et al*, 2012). For instances, in mammals, the exon has been suggested as the primary evolutionary unit, while the intron was considered as the unit in *Drosophila* (Xiao *et al*, 2007). Moreover, the cotranscriptional splicing efficiency also differs dramatically between *Drosophila* and mouse (Khodor *et al*, 2012). Other explanations could also be (i) the conclusion in the *Drosophila* study might be affected by a much lower number of divergent events identified there (between *Drosophila melanogaster* and *Drosophila simulans*, seven and four divergent SE were attributed to *cis-* and *trans-*divergence, whereas between *Drosophila melanogaster* and *Drosophila sechellia*, two and three divergent SE were attributed to *cis-* and *trans-*effects, respectively). (ii) The study designs were different (whole animal for *Drosophila* versus distinct cell/tissue for mouse).

*Cis*-regulatory divergence results solely from sequence variants in pre-mRNA sequences, which could affect directly canonical splicing sites or exonic/intronic regulatory elements. Among the *cis-*divergent events identified in this study, 41.4% contained sequence variants at the canonical splice sites, a proportion of which could substantially affect the strength of splicing sites. The remaining events without sequencing variants at splicing sites could be used to identify potential exonic/intronic regulatory variants, as demonstrated in this study. Using the same F1 hybrid mice, future datasets on the allelic splicing obtained from different tissues could be used to discover more novel regulatory elements, especially tissue-specific ones.

## Materials and Methods

### Mouse liver sample collection and fibroblast cell culture

SPRET/EiJ mice were purchased from The Jackson Laboratories (Maine, USA), and C57BL6/J mice were obtained from Janvier (Le Genest-Saint-Isle, France). Both mouse strains were bred further in our animal house (VIB and Ghent University). C57BL6/J females were crossed with SPRET/EiJ males to yield F1(BxS) hybrid mice. All mice were kept in an air-conditioned, temperature-controlled conventional animal house and obtained food and water *ad libitum*. Mice were used at the age of 8 weeks. All animal husbandry and experiments were approved by the local ethical committee (VIB and Ghent University). Mice were killed by acute CO intoxication, and livers were excised under sterile conditions. Livers were snap-frozen in liquid nitrogen and kept at −80°C until further use.

Adult mouse fibroblast cells were isolated and cultured according to the protocol from ENCODE project (http://genome.ucsc.edu/ENCODE/protocols/cell/mouse/Fibroblast_Stam_protocol.pdf) with modification of cell culture medium (RPMI 1640 Medium, GlutaMAX™ Supplement (Gibco, Life Technologies) with 10% FBS and 1% P/S). F1(BxS) mice used for fibroblast cell isolation were obtained as described before (Gao *et al*, 2013).

### RNA sequencing

Total RNAs were extracted using TriZOL reagent (Life Technologies) following manufacturer’s protocol. Stranded mRNA sequencing libraries were prepared with 500 ng total RNA according to manufacturer’s protocol (Illumina). The libraries were sequenced in 2 × 100nt + 7 manner on HiSeq 2000/2500 platform (Illumina).

### Reference sequences and gene annotation

The reference sequences and the Ensembl gene annotation of the C57BL/6J genome (mm10) were downloaded from the Ensembl FTP server (ftp://ftp.ensembl.org, version GRCm38, release 74). The SNVs and indels between C57BL/6J and SPRET/EiJ were downloaded from Mouse Genome Project Web site (http://www.sanger.ac.uk/). The vcf2diploid tool (version 0.2.6) in the AlleleSeq pipeline was used to construct the SPRET/EiJ genome by incorporating the SNVs and indels into the C57BL/6J genome (Rozowsky *et al*, 2011). The chain file between the two genomes was also reported as an output, which was further used with the UCSC liftOver tool.

### RNA-seq read preprocessing and alignment

Flexbar was first used to trim the RNA-seq reads that pass the Illumina filter to remove library adapter sequences with parameters -f i1.8 -x 6 -u 0 -m 90 -k 90 -ae RIGHT (Dodt *et al*, 2012). Here, in addition to the adapter sequences, we trimmed the first six bases on the 5′ end to remove the sequence artifact due to the use of random hexamer as RT primers (-x 6). We retained only the read pairs with both reads of length ≥ 90 nucleotides after adapter removal (-m 90) and trimmed all of them from 3′ end to the same length of 90 nucleotides (-k 90).

The remaining RNA-seq reads were aligned to the mouse genomes’ reference sequences (see above) using TopHat with default mapping parameter and Ensembl gene annotation (version 2.0.8) (Trapnell *et al*, 2009). For RNA-seq samples from parental strains, reads were aligned to the corresponding genome. For mixed (mock F1 hybrid) and F1 hybrid samples, reads were first aligned to both genomes and then assigned to the parental allele with less mapping edit distance. The reads with equal mapping distance to both genomes were discarded, and only, the allele-specific reads were retained for further analysis. Genomic alignment coordinates for SPRET/EiJ were then converted to the corresponding locations in the C57BL/6J reference genome using the UCSC liftOver tool and their chain files.

### Alternative splicing analysis

Mixture of Isoforms (MISO) Bayesian Inference model (version 0.4.9) was used for quantification and comparison of alternative splicing events (Katz *et al*, 2010). The MISO algorithm counts the numbers of reads that are common to both isoforms and the reads that are exclusive to one isoform or the other, in order to estimate the percent spliced-in (PSI) values in a given sample. The MISO events database (mm10) was downloaded from the MISO Web site (http://genes.mit.edu/burgelab/miso). Only the events from autosome were considered in this study. Splicing analysis was performed for the events supported with at least 20 RNA-seq reads (spliced-in + spliced-out) in all the replicate samples.

The Bayesian factor (BF) was used as a measure of statistical significance for PSI difference. Based on prior work, BF > 5 in all the replicates and average |ΔPSI| > 0.1 was used as the threshold for determining significant splicing difference between two parental strains or two alleles. To check whether our conclusion was sensitive to different thresholds, we also tried different cutoffs of |ΔPSI| values (|ΔPSI|> 0.0, 0.05, and 0.15, respectively) corresponding to different FDRs (See False discovery rate estimation section for details, and FigEV1).

*Trans*-regulatory divergence in alternative splicing was estimated using the method of Altman and Bland (Altman & Bland, 2003; McManus *et al*, 2014). In brief, the ratio of PSI values between strains was compared to allele-specific PSI ratios from F1 hybrid. The standard error of the difference in parental and allelic PSI ratios was calculated and used to derive *Z*-scores and *P*-values. *Q*-values were further calculated using the “qvalue” module in R, and a same FDR cutoff as for *cis*-regulatory divergence was applied to determine *trans*-regulatory splicing divergence (Storey & Tibshirani, 2003).

### False discovery rate estimation

To estimate the FDR, we used a method based on bootstrapped label permutation, as described before (Sterne-Weiler *et al*, 2013). In brief, for each value of *x* from 0.01 to 0.20 increasing by 0.01, we performed independent 100 bootstrapped label permutations of other replicates. For each of the 100 shuffled sets, we calculated the number of events passing the threshold (false positives), *that is* BF > 5 in all the replicates and average |ΔPSI| > *x*. Then, for each of the 100 permutations of each value *x*, the FDR was estimated as false positives divided by the number of real events passing the threshold, including both false positives and true positives.

### Filter with mock F1 hybrid

In F1 hybrid, only the reads that could be unambiguously assigned to either genome were retained for the estimation of alternative splicing (see RNA-seq read preprocessing and alignment section). Therefore, the events with low variation density could have low coverage in F1 hybrid sample, or inconsistent PSI values between the parental strains and their F1 hybrid. To avoid potential errors, we mixed C57BL/6J reads and SPRET/EiJ reads to create mock F1 hybrid samples, which were then processed in the same way as the real F1 hybrid samples (i.e. mapping to both genomes and assignment to the parental alleles for the identification of allele-specific reads according to edit distance). To evaluate the variations of PSI values for the events without assignment bias, we also downsampled the C57BL/6J reads to the same coverage as the C57BL/6J allele in mock F1 hybrid and then mapped these reads to C57BL/6J genome, and likewise for SPRET/EiJ reads.

To detect the events with inconsistent PSI values between the parental strains and the mock F1 hybrid, we applied a *Z*-value transformation, *that is* ΔPSI (the difference between the PSI values and the mock F1 hybrid PSI values) by a local standard deviation which we computed using a sliding window approach as following. In the downsampled data, after sorting the events according to the total number of spliced-in and spliced-out reads used for computing the PSI values, we calculated for each data point the standard deviation of the respective values inside a window consisting 1% events. The local standard deviations were then smoothed using loess regression before we used them for calculating *Z*-values and *P*-values in mock F1 hybrid sample. *P*-values were then adjusted using Benjamini–Hochberg method, and a false discovery rate of 0.05 was applied to filter out the events with inconsistent PSI values.

### RT–PCR and PacBio sequencing

Starting from 5 ug total RNA, polyA RNA was enriched using Dynabeads oligo-dT beads (Life Technologies), and reverse transcription (RT) was performed using random hexamer and SuperScript II reverse transcriptase. PCR was followed using 1 μl of RT product as template in 50 μl of GoTaq PCR system (Promega). PCR primers were designed for amplifying the genomic region covering the alternative splicing events (Table EV3). PCR program was as follows: 4 min at 95°C; followed by 28 cycles of 30 s at 95°C, 30 s at 55°C, and 45 s at 72 °C; and a final elongation of 10 min at 72°C. Different PCR products from the same RT product using different primers were then mixed and purified using Agencourt AMPure XP system (Beckman Coulter) and quantified by Qubit HS dsDNA measurement system (Life Technology). These mixed PCR products were then sequenced on PacBio RS SMRT platform according to the manufacturer’s instruction.

Sequence reads from the PacBio RS SMRT chip were processed through PacBio’s SMRT-Portal analysis suite to generate circular consensus sequences (CCSs). The CCSs were then mapped to a reference database containing alternative splicing isoforms from both alleles using BLAST with default parameters. The best hit was retained for each aligned sequence read. The reads with multiple best hits were discarded. PSI values were calculated as No. long-isoform-supporting-reads/(No. long-isoform-supporting-reads + No. short-isoform-supporting-reads).

### C57BL/6J, CAST/EiJ, and their F1 hybrid liver data analysis

The C57BL/6J, CAST/EiJ, and their F1 hybrid liver data were downloaded from previous study and processed in the same way as our data. Due to lower sequencing depth and lower density of sequence variants between these two strains, we pooled their dataset into two replicates for C57BL/6J, CAST/EiJ, and their F1 hybrid, respectively. Specifically, ERR185942, ERR185943, and ERR120684 were pooled into C57BL/6J replicate 1; ERR120686, ERR120702, and ERR120704 were pooled into C57BL/6J replicate 2; ERR120692, ERR120694, and ERR120698 were pooled into CAST/EiJ replicate 1; ERR185946, ERR185947, and ERR185948 were pooled into CAST/EiJ replicate 2; ERR120672, ERR185940, ERR185941, ERR120678, ERR185945, and ERR120700 were pooled into F1 hybrid replicate 1; ERR185944, ERR120696, ERR185949, ERR185950, ERR185951, and ERR185952 were pooled into F1 hybrid replicate 2.

### Control events without *cis*-regulatory divergence

To compare with the events with *cis*-regulatory divergence, we selected a separate group of AS events that passed the minimum threshold of 20 supporting reads but did not show splicing divergence between the two strains (BF < 1 and 0.05 < PSI < 0.95 in all three replicates as well as average |ΔPSI| < 0.05).

### Splicing site strength score analysis

For each splicing event, the nucleotide sequences of 5′ and 3′ splice sites were first extracted from the C57BL/6J and SPRET/EiJ genomes according to their locations (in.fasta format). These sequences were then uploaded to the “Analyzer Splice Tool” server (http://ibis.tau.ac.il/ssat/SpliceSiteFrame.htm) to calculate the splicing site strength score. For SE, RI, and MXE, the strength scores of 5′ and 3′ splice site were combined.

### Five mouse strains brain data analysis

The C57BL/6NJ, PWK/PhJ, WSB/EiJ, CAST/EiJ, and SPRET/EiJ brain data were downloaded from previous study (accession number: ERP000614) (Danecek *et al*, 2012), and then, MISO (version 0.4.9) was used for the quantification of alternative splicing events in each dataset.

### Minigene plasmids’ construction and *in vitro* minigene splicing reporter assay

Two C57BL/6J homologue genomic regions from Trim26 gene were amplified from 100 ng of C57BL/6J genomic DNA using 50 μl of Phusion PCR system (Thermo Scientific), respectively, with PCR program of 3 min at 98°C; followed by 40 cycles of 30 s at 98°C, 30 s at 57°C, and 1 min at 72°C; and a final elongation of 10 min at 72°C. For the PCR of the first C57BL/6J homologue genomic region, the PCR primers were designed as follows: one targeting on exon 1 (MG1-1-F: AAGCTGGCTAGCGTTTAAACTTAAGCTTGCTTGCTCAGGACCTACCCCGCGG); the other targeting on the region from the exon 2 to the adjacent region in intron 2 with four versions containing different combinations of SPRET/EiJ variants, respectively, (MG1-1-no_variant-R: TAAACAGATACATAAATATAAGACCTGCTTCTGGTCATGCAGGGCTCCAAGCCACCAGGTGGAACGTCATCCGGGTC; MG1-1-insert-R: TAAACAGATACATAAATATAAGACCTGCTTCTGGTCATGCAGGGCTCCAAGCCCAAGCTCCAACCAGGTGGAACGTCATCCGGGTC; MG1-1-SNV-R: TAAACAGATACATAAATATAAGACCTGCTTCTGGTCATGCAGGGCTCCAAGCCAGCAGGTGGAACGTCATCCGGGTC; MG1-1-SNV_insert-R: TAAACAGATACATAAATATAAGACCTGCTTCTGGTCATGCAGGGCTCCAAGCCCAAGCTCCAAGCAGGTGGAACGTCATCCGGGTC). For the PCR of the second C57BL/6J homologue genomic region, the PCR primers were designed as follows: one targeting on intron 2 region adjacent to exon 3 with 5′ overhang sequence overlapping with intron 2 part of the first PCR product (MG1-2-F: GCAGGTCTTATATTTATGTATCTGTTTATTTTTTTTTTATTTATTTATCCTCAGAGTCATAGCCCGGGACAGCCACAGAGGA); the other targeting on exon 3 (MG1-2-R: TCTAGACTCGAGCGCGGATCCATATGGGGCGGATATCACTTGTGCAG). The PCR products from above were purified using Agencourt AMPure XP system (Beckman Coulter). Then, the overlapping PCR was performed between 15 ng of PCR products from the first and second Trim26 genomic regions using 50 μl of Phusion PCR system (Thermo Scientific) with PCR program of 3 min at 98°C; followed by eight cycles of 30 s at 98°C, 30 s at 55°C, and 1 min at 72°C, then adding 10 nmol of MG1-1-F and MG1-2-R primers; followed by 27 cycles of 30 s at 98°C, 30 s at 55°C, and 1 min at 72°C; and a final elongation of 10 min at 72°C. Overlapping PCR products were purified using Agencourt AMPure XP system (Beckman Coulter), cut by NheI and XhoI restrict enzymes (NEB), and subcloned into pcDNA3.1/Hygro(+) vector (Invitrogen). Final minigene constructs were sequenced to verify the sequences and variants.

HEK293T and NIH3T3 cell lines (ATCC) were grown in DMEM (Invitrogen) with 10% FBS (Invitrogen). Cells were plated in 6-well plates and transfected using Lipofectamine 2000 (Invitrogen) according to manufacturer’s protocol. Total RNAs were purified 48 h after transfection using TriZOL reagent (Invitrogen) and reverse-transcribed into ss-cDNA using oligo-dT primer with SuperScript II reverse transcription system (Invitrogen). PCR was then performed using 50 μl of GoTaq PCR system with 1 μl of cDNA, 10 nmol of PCR primers T7-Promoter (TAATACGACTCACTATAGGG) and BGH-reverse (TAGAAGGCACAGTCGAGG), and PCR program of 2 min at 95°C; followed by either 25 cycles (HEK293T) or 40 cycles (NIH3T3) of 30 s at 95°C, 30 s at 54°C, and 1 min at 72°C; and a final elongation of 10 min at 72°C. Amounts of RT–PCR products were measured by Bioanalyser DNA 1000 chip (Agilent).

### Data access

The RNA-seq data from this publication have been submitted to the European Nucleotide Archive (http://www.ebi.ac.uk/ena) and assigned the accession ERP006913.

## Supporting Information

**Expanded View Figures PDF**

**Table EV1**

**Table EV2**

**Table EV3**

**Table EV4**

**Review Process File**
