# Supplementary material for: Predominant contribution of cis-regulatory divergence in the evolution of mouse alternative splicing
Source: Mol Syst Biol. 2015 Jul 1;11(7):816. doi: 10.15252/msb.20145970 (PMC4547845; doi:10.15252/msb.20145970)
Supplement: Supplementary file 1 [file msb0011-0816-sd1.pdf]

Expanded View Figures

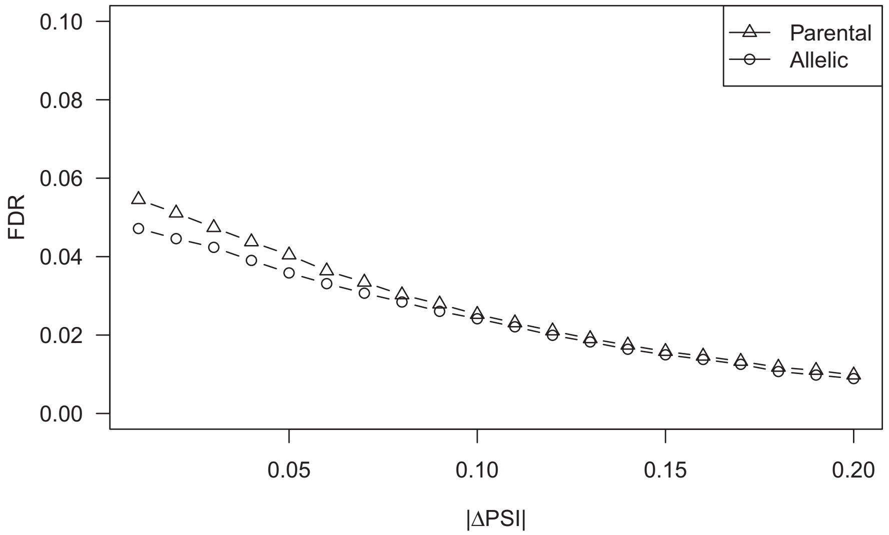

Figure EV1. FDR estimation for each |ΔPSI| cutoff.
FDR for parental (triangle) and allelic (circle) splicing comparison (*y*-axis) was plotted against different |ΔPSI| cutoffs (*x*-axis). For each value of *x* from 0.01 to 0.20 increasing by 0.01, we performed independent 100 bootstrapped label permutations of replicate 2 and replicate 3, respectively. For each of the 100 shuffled sets, we calculated the number of events passing the threshold (false positives), *that is* BF > 5 in all the replicates and average |ΔPSI| > *x*. Then, for each of the 100 permutations of each value *x*, the FDR was estimated as false positives divided by the number of real events passing the threshold, including both false positives and true positives.

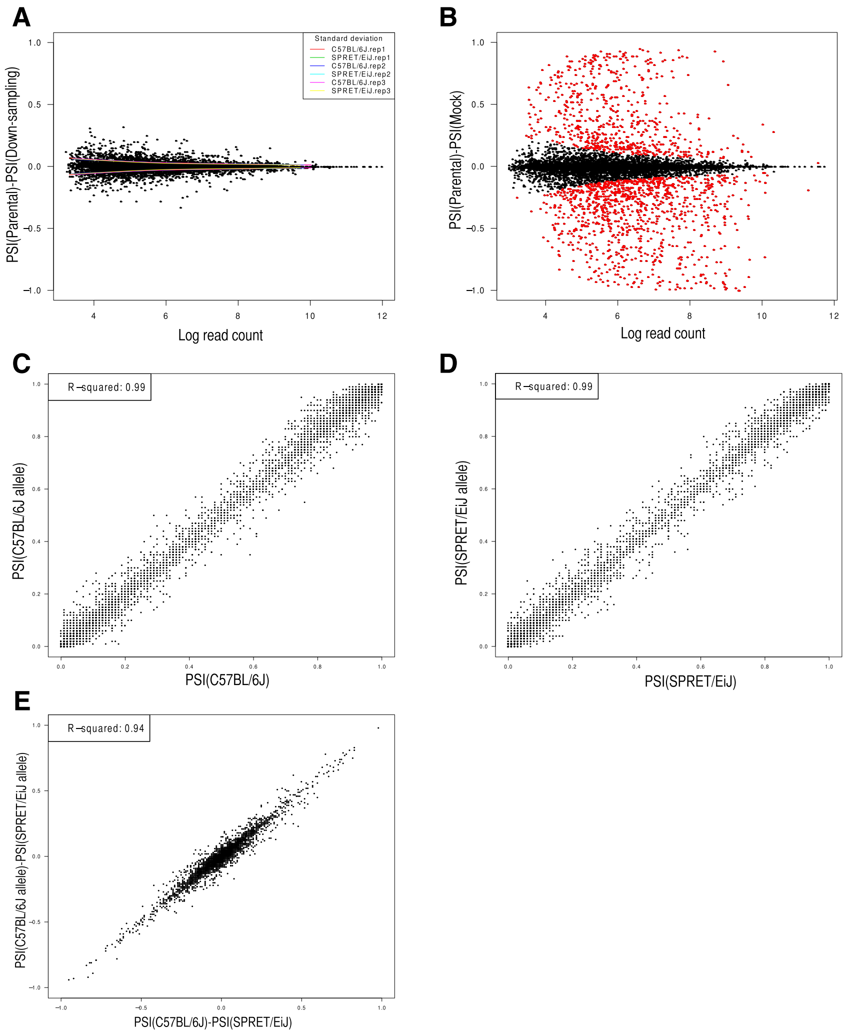

Figure EV2. Illustration of data filtering based on mock F1 hybrid.

- A MA plot comparing the PSI values in parental strains and their downsampling datasets. The local standard deviation for each comparison was also indicated (see Materials and Methods).
- B MA plot comparing the PSI values in parental strains and those estimated based on mock F1 dataset. The red dots represented the outliers with inconsistent PSI values between parental strain and mock F1 dataset.
- C–E After filtering, the PSI values for C57BL/6J (C), SPRET/EiJ (D), and their difference (E) correlated well between parental strains and mock F1 hybrid (R^2 = 0.99, 0.99 and 0.94, respectively).

© 2015 The Authors

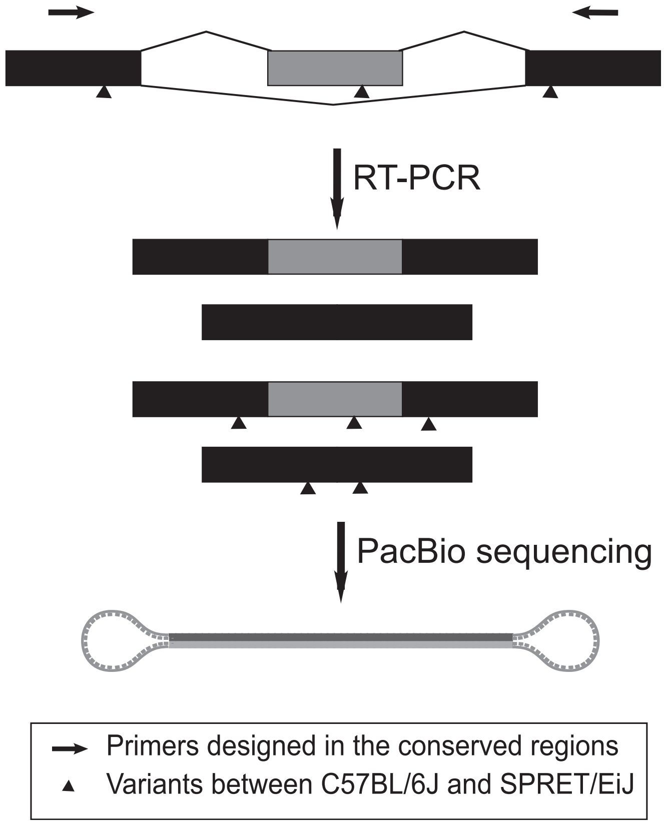

Figure EV3. Illustration of PacBio sequencing of splicing event-spanning cDNA products.
For each candidate event, RT–PCR primers were designed in the conserved regions of the constitutive exons to amplify both isoforms from the two alleles/strains. The PCR products were then sequenced at full length using PacBio RS system.

 © 2015 The Authors

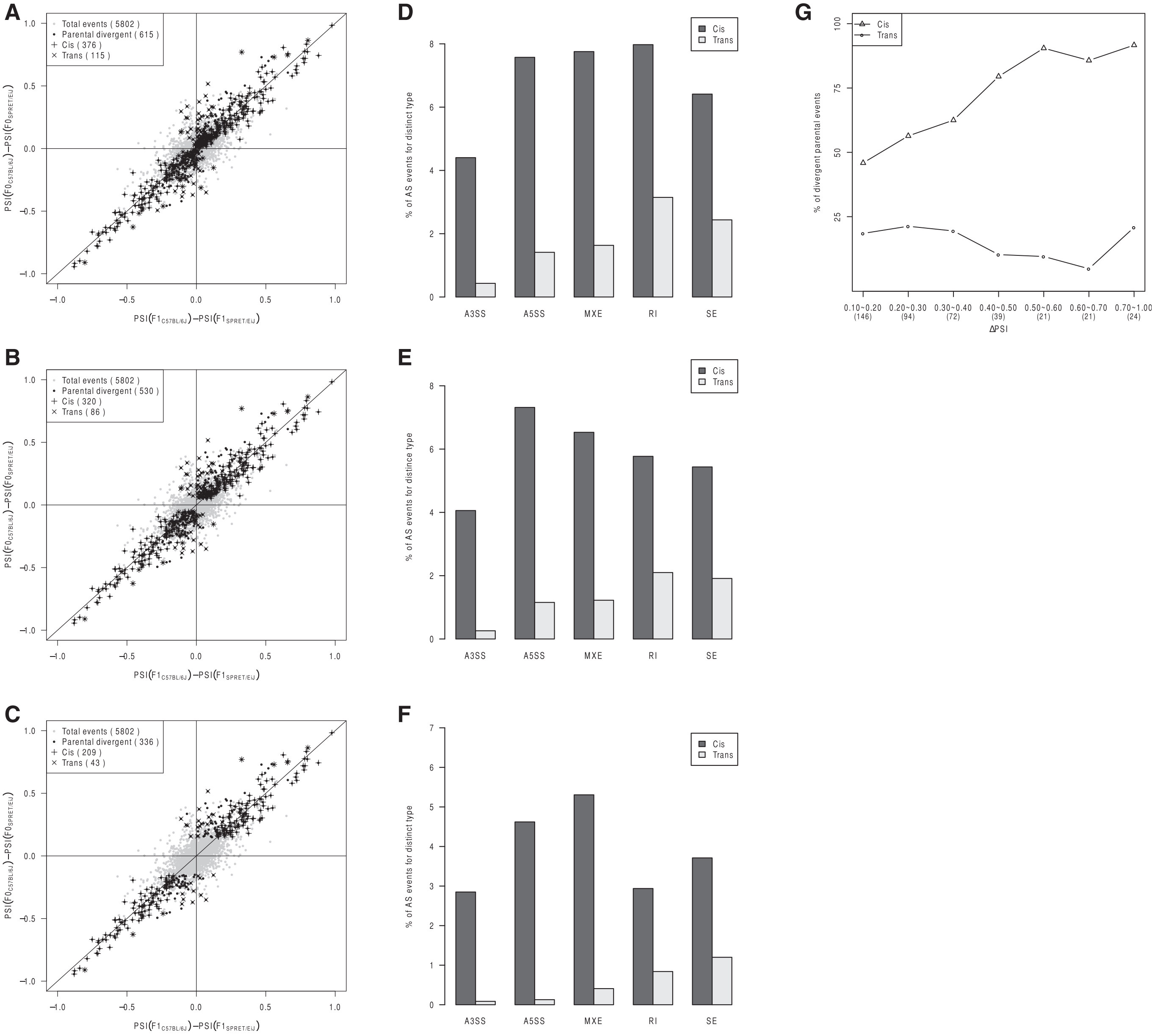

Figure EV4. Dissection of *cis*- and *trans*-regulatory contributions in alternative splicing at different |ΔPSI| cutoffs.

A–C Scatterplot comparing splicing differences in parental strains (*y*-axis) versus the allelic differences in F1 hybrid (*x*-axis) at different |ΔPSI| cutoffs [|ΔPSI| > 0 (A), 0.05 (B) and 0.15 (C)]. After filtering using mock F1 hybrid, 5,802 AS events were expressed in F1 hybrid (gray dots). Among these, 615 (A)/530 (B)/336 (C) AS events were divergent between parental strains (black dots), of which 376 (A)/320 (B)/209 (C) (indicated as "+") and 115 (A)/86 (B)/43 (C) (indicated as "×") exhibited significant *cis*- and *trans*-regulatory divergence, respectively.

D–F Percentage of *cis*- and *trans*-divergent events for the five AS types separately at different |ΔPSI| cutoffs [|ΔPSI| > 0 (D), 0.05 (E), and 0.15 (F)].

G Contributions of *cis* (indicated as triangle)-/*trans* (indicated as circle)-regulatory divergence (*y*-axis) to parental divergent AS events with different effect sizes (|ΔPSI|, *x*-axis). A total of 417 divergent events between parental strains (see Fig 2B) were grouped into 7 categories according to the |ΔPSI| values: (0.1, 0.2), (0.2, 0.3), (0.3, 0.4), (0.4, 0.5), (0.5, 0.6), (0.6, 0.7), and (0.7, 1.0). The number of events in each category was marked. While *cis*-regulatory divergence always played the predominant role in determining parental AS divergence with different effect sizes, its relative contribution slightly decreased with the decreasing effect size.

© 2015 The Authors

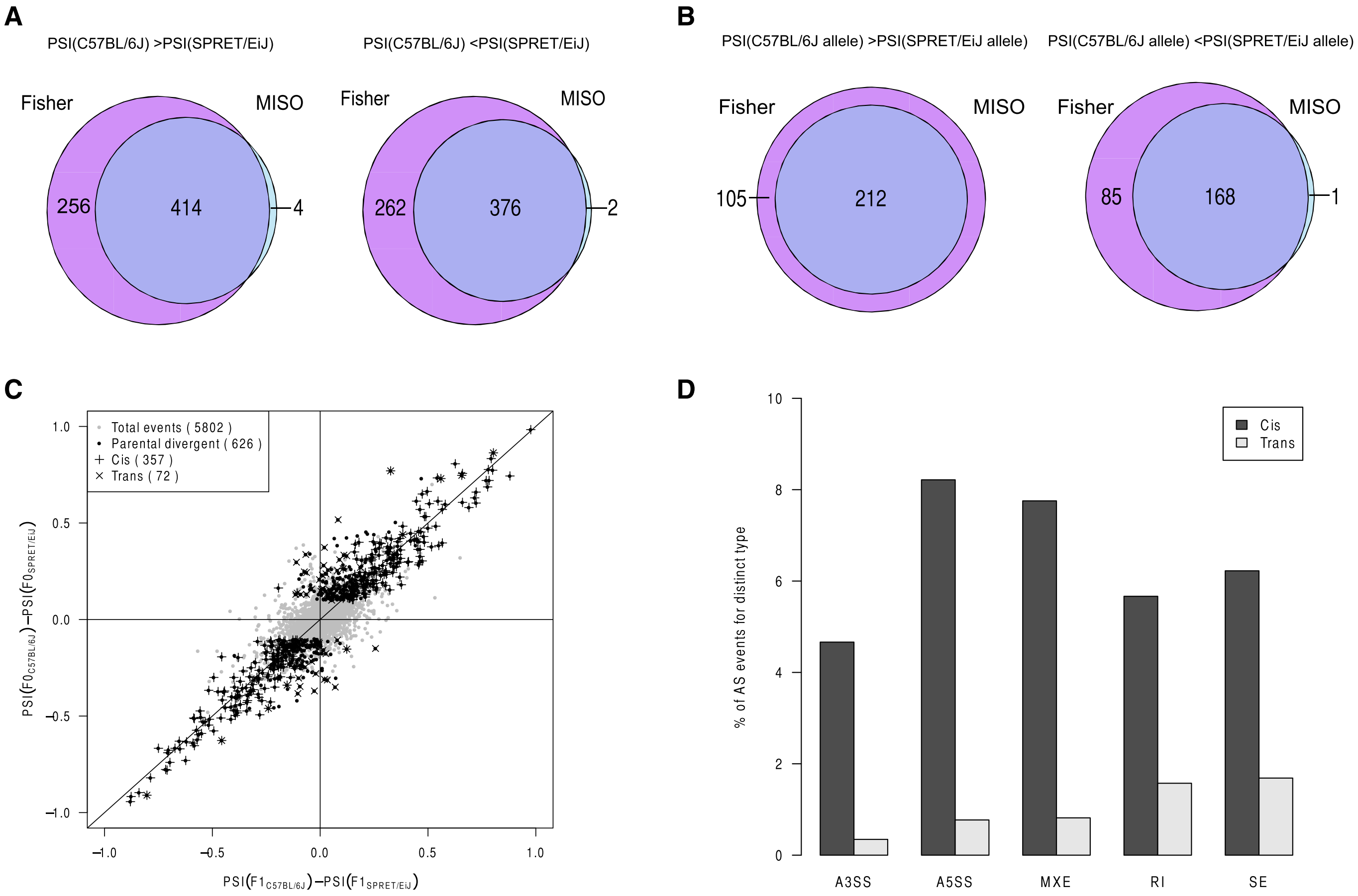

Figure EV5. Divergent AS events identified using Fisher's exact test.

A, B Venn diagram showing the overlap of the divergent events identified by Fisher's exact test and MISO in parental strains (A) and in F1 hybrid (B).

C Scatterplot comparing splicing difference in parental strains versus the allelic difference in F1 hybrid identified by Fisher's exact test. After filtering using mock F1 hybrid, 5,802 AS events were expressed in F1 hybrid (gray dots). Among these, 626 AS events were divergent between parental strains (black dots), of which 357 (indicated as "+") and 72 (indicated as "×") exhibited significant *cis*- and *trans*-regulatory divergence, respectively.

D Percentage of *cis*- and *trans*-divergent events for the five AS types separately using Fisher's exact test.

© 2015 The Authors

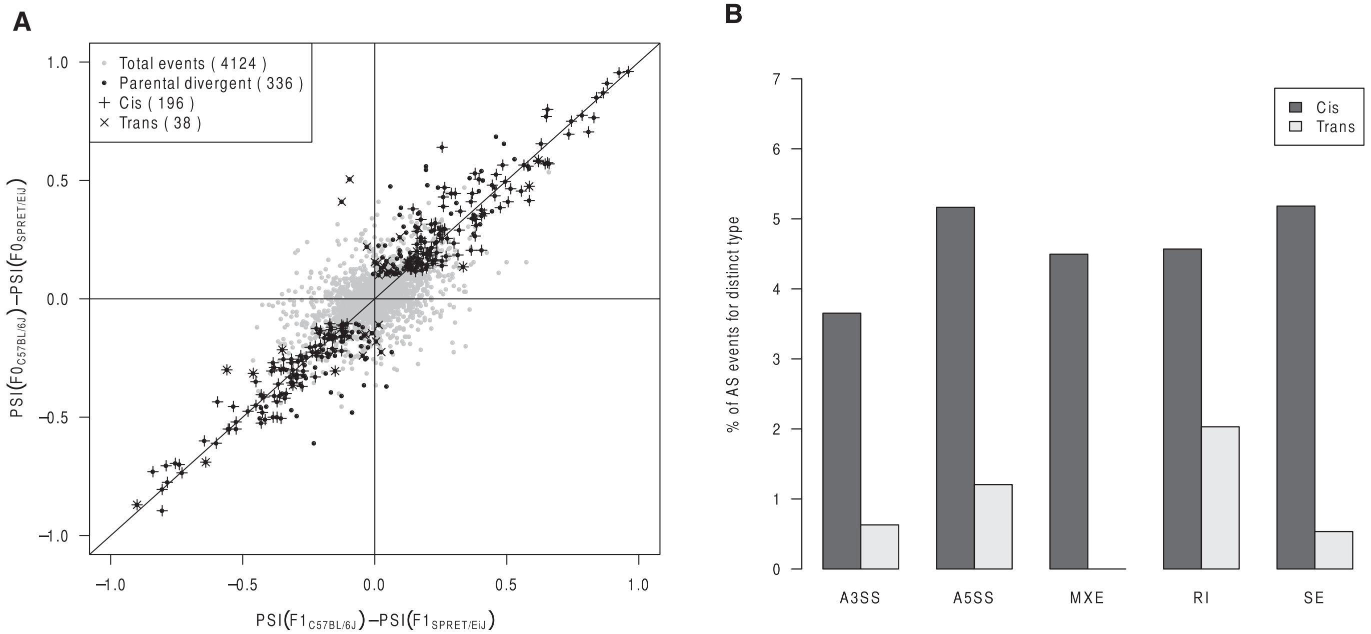

Figure EV6. Dissection of *cis*- and *trans*-regulation in alternative splicing between C57BL/6J and SPRET/EiJ liver samples.

A Scatterplot comparing splicing difference between C57BL/6J and SPRET/EiJ liver samples versus their allelic difference in F1 hybrid liver sample. After filtering using mock F1 hybrid, 4,124 AS events were expressed in F1 hybrid (gray dots). Among these, 336 AS events were divergent between parental strains (black dots), of which 196 (indicated as “+”) and 38 (indicated as “×”) exhibited significant *cis*- and *trans*-regulatory divergence, respectively.

B Percentage of *cis*- and *trans*-divergent events for the five AS types separately.

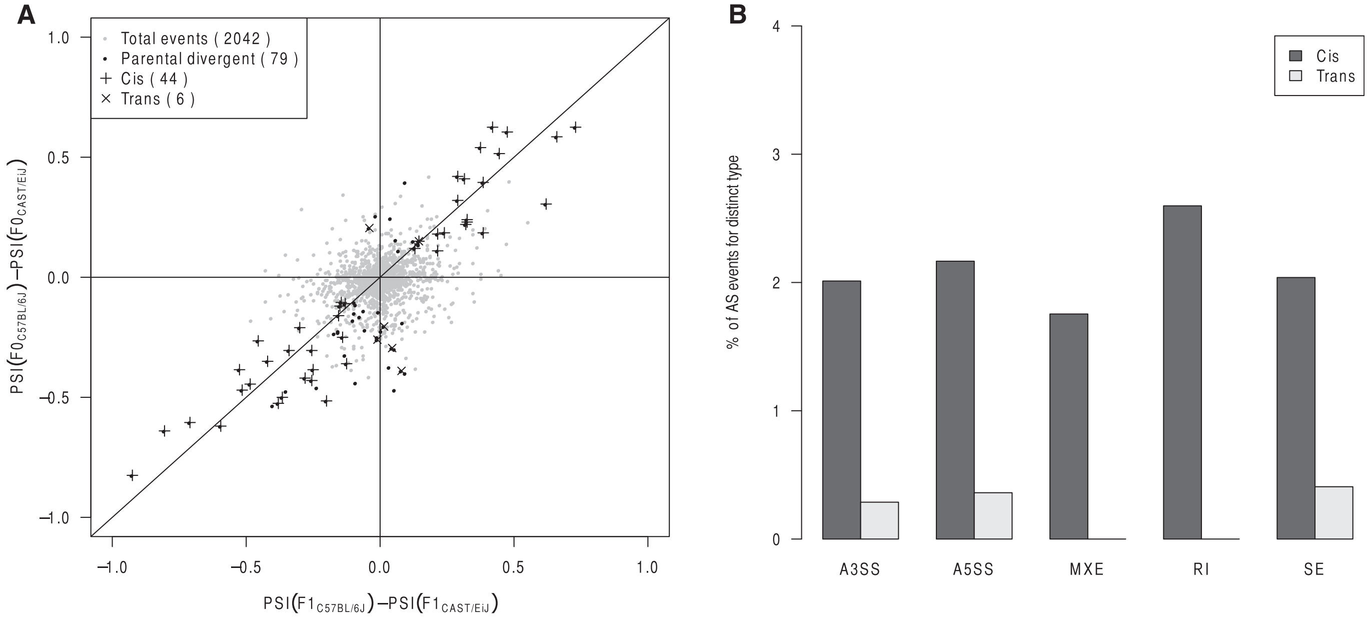

Figure EV7. Dissection of *cis*- and *trans*-regulation in alternative splicing between C57BL/6J and CAST/EiJ.

A Scatterplot comparing splicing difference between C57BL/6J and CAST/EiJ versus their allelic difference in F1 hybrid. After filtering using mock F1 hybrid, 2,042 AS events were expressed in F1 hybrid (gray dots). Among these, 79 AS events were divergent between parental strains (black dots), of which 44 (indicated as “+”) and 6 (indicated as “×”) exhibited significant *cis*- and *trans*-regulatory divergence, respectively.

B Percentage of *cis*- and *trans*-divergent events for the five AS types separately.

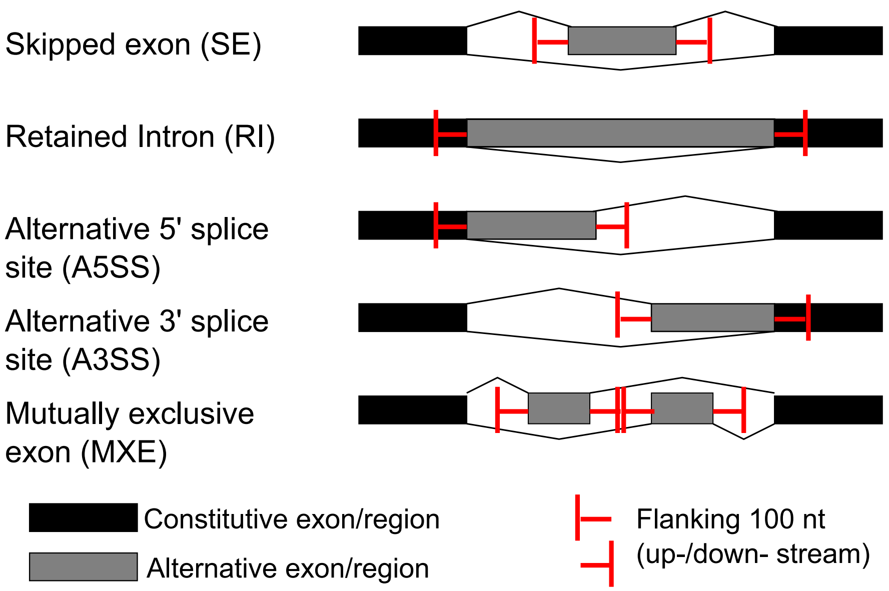

Figure EV8. Illustration of the regions flanking the AS events.
For SE, the alternative exons and their flanking 100 nt intron sequences were considered; for RI, the retained introns and their flanking 100 nt exon sequences were considered. For A3SS or A5SS, the alternative exon regions and their flanking 100 nt exon/intron sequences were considered. For MXE, both alternative exons and their flanking 100 nt intron sequences were considered.

© 2015 The Authors

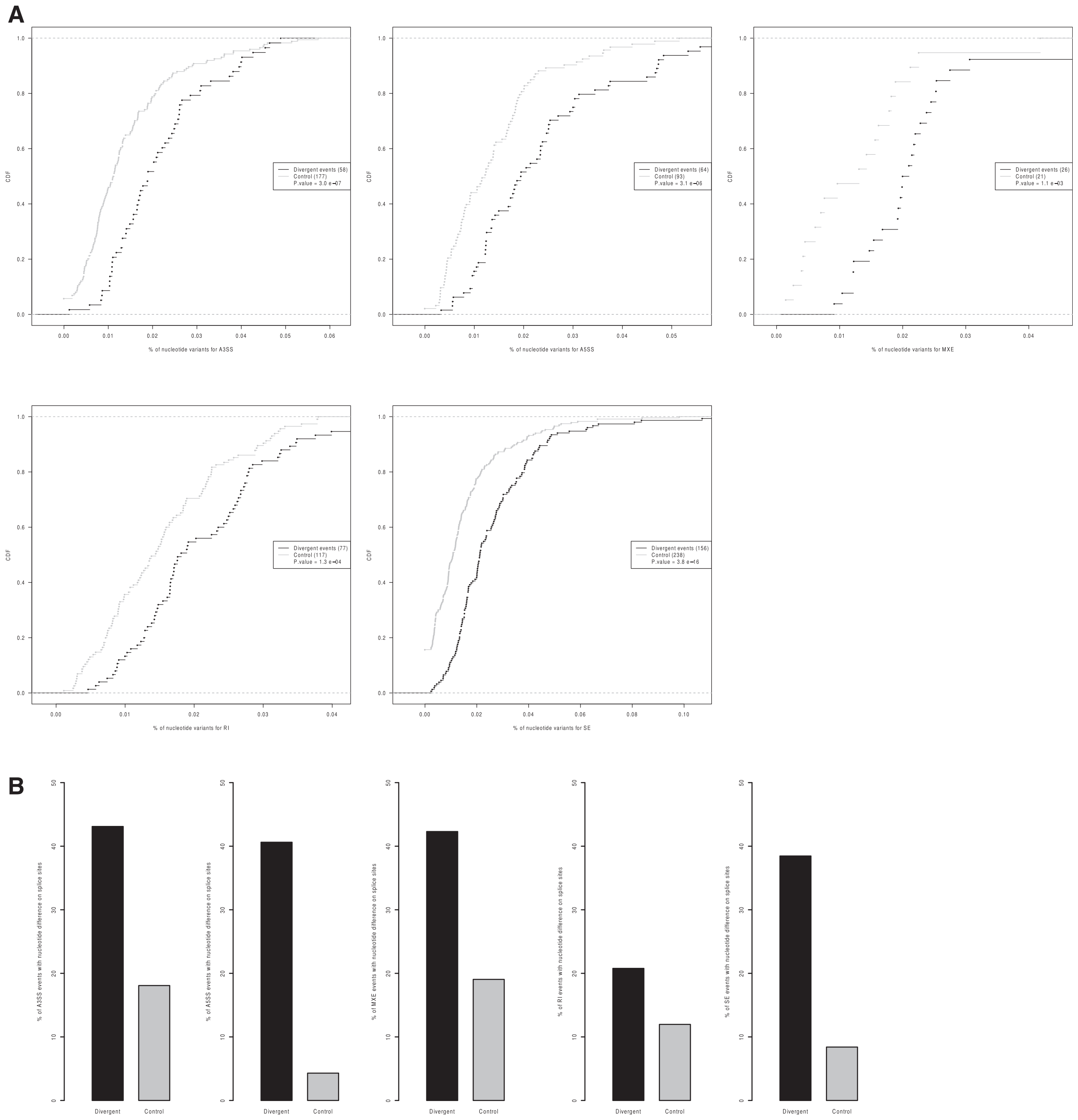

Figure EV9. Genomic features that correlate with *cis*-regulatory alternative splicing divergence for each AS type separately.

A CDF of frequencies of nucleotide variants in the AS flanking regions for the events with *cis*-regulatory divergence (black) and controls (grey) for A3SS, A5SS, MXE, RI, and SE, respectively.

B Percentages of the events with significant *cis*-regulatory divergence (black) and controls events (gray) that had sequence divergence at the exact splice sites for A3SS, A5SS, MXE, RI, and SE, respectively.

© 2015 The Authors

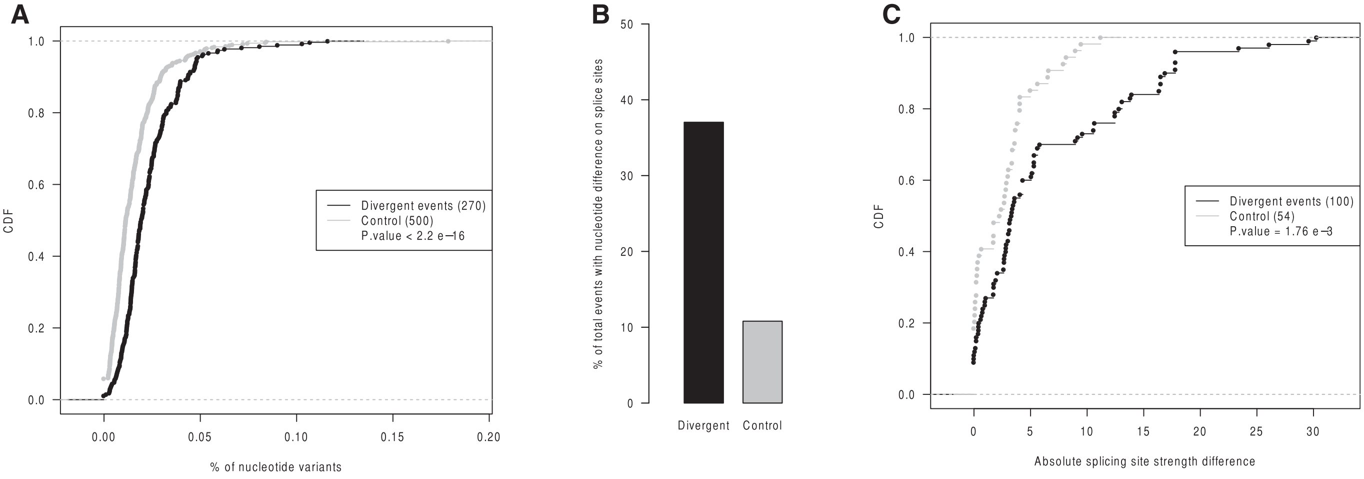

Figure EV10. Genomic features that correlate with *cis*-regulatory alternative splicing divergence identified in the liver sample.

A CDF of frequencies of nucleotide variants in the AS flanking regions for the events with *cis*-regulatory divergence (black) and controls (grey) identified in liver sample. Compared with controls, the events with significant *cis*-regulatory impact also had higher sequence divergence in the flanking regions.

B In liver sample, 37.0 and 10.8% of the events with significant *cis*-regulatory divergence (black) and controls (gray) had sequence divergence at the exact splice sites, respectively.

C CDF of allelic differences in splicing site strengths due to sequence variants at the exact splicing sites plotted for *cis*-regulatory divergent events (black) and controls (grey) identified in liver sample. The splicing site strengths changed more in the events with *cis*-regulatory events than in those without.

 © 2015 The Authors

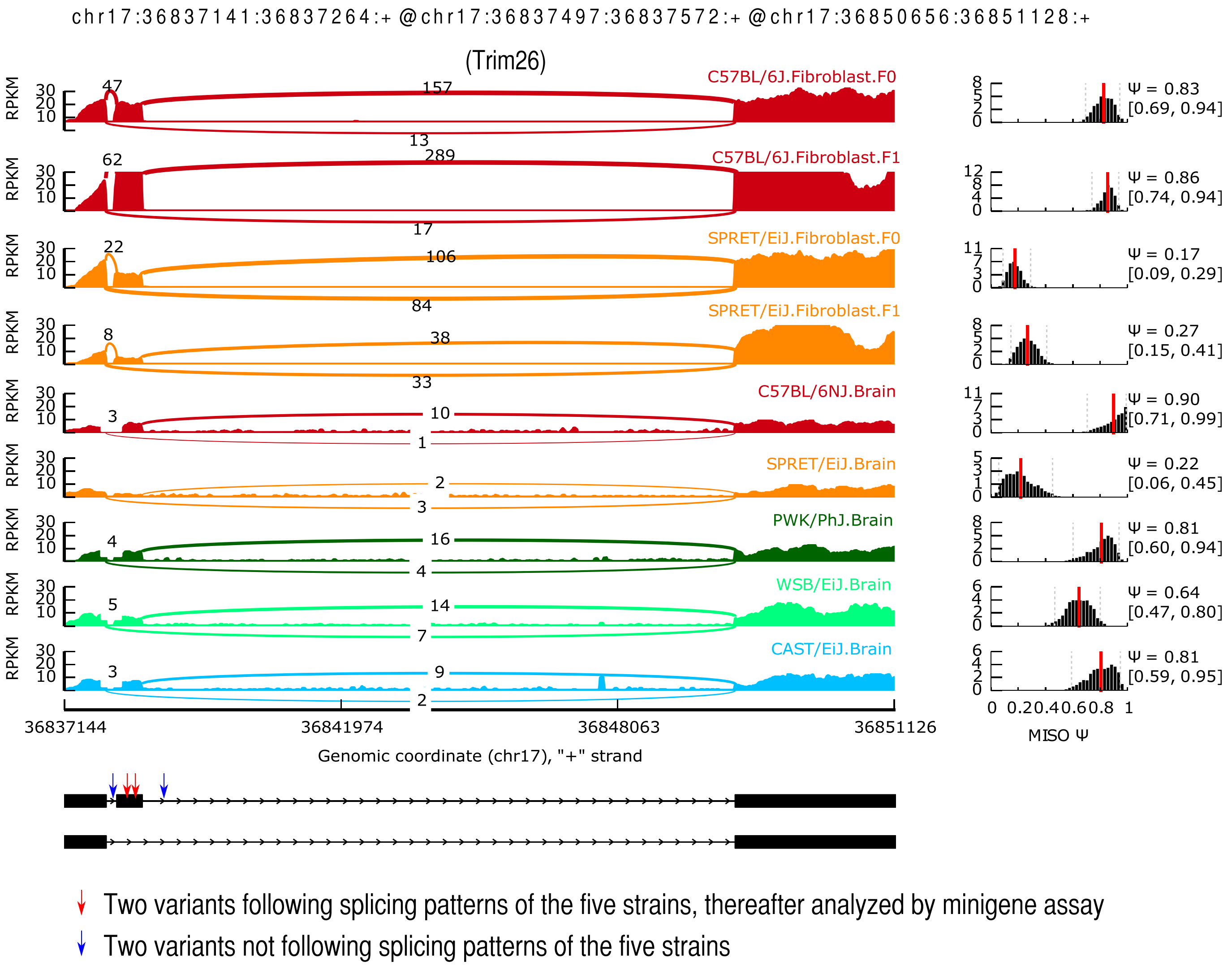

Figure EV11. Sashimi plot for the splicing patterns of the SE event in Trim26 gene from fibroblast cell line as well as brain tissues of five mouse strains.
The top four rows represented splicing patterns for C57BL/6J and SPRET/EiJ strains and their alleles in F1 hybrid. The bottom five rows represented splicing patterns for brains tissues of the five mouse strains. PWK/PhJ and CAST/EiJ had a similar splicing pattern as C57BL/6J, but different from SPRET/EiJ. Four variants located in the flanking regions, two of which correlated with the species-specific splicing pattern and were analyzed using minigene assays (see Fig 4).

© 2015 The Authors

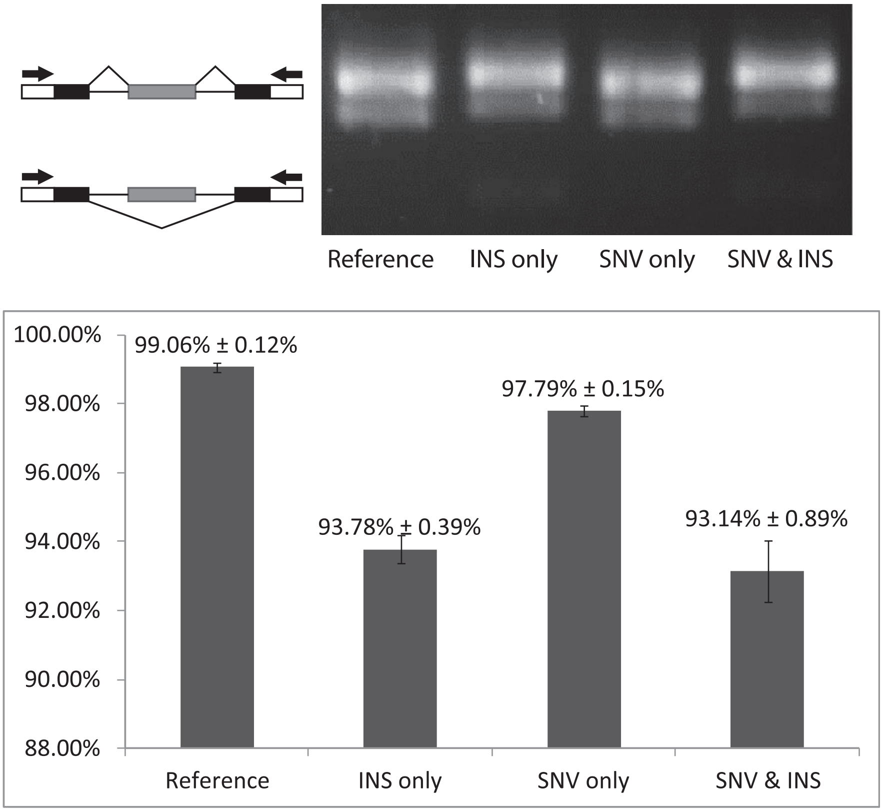

Figure EV12. Minigene analysis for the *cis*-divergent SE event in Trim26 gene in NIH3T3 cells.
Label is the same as in Fig 4B.

 © 2015 The Authors
