# Supplementary material for: Predominant contribution of cis-regulatory divergence in the evolution of mouse alternative splicing
Source: Mol Syst Biol. 2015 Jul 1;11(7):816. doi: 10.15252/msb.20145970 (PMC4547845; doi:10.15252/msb.20145970)
Supplement: Supplementary file 2 [file msb0011-0816-sd2.doc]

Table EV1 - Summary of sequencing results

| Samples | | | | Total read pairs  [million] | Read pairs after trimming  [million] | Concordantly mapped read pairs [million (%)]^1)^ | Assigned to allelic origin  [million (%)]^2)^ |
| --- | --- | --- | --- | --- | --- | --- | --- |
| Fibroblast cell line | C57BL/6J | | Replicate 1 | 168.6 | 150.5 | 129.4 (86.0%) |  |
|  |  |  | Replicate 2 | 156.0 | 137.7 | 120.2 (87.3%) |  |
|  |  |  | Replicate 3 | 175.0 | 156.1 | 133.3 (85.4%) |  |
|  | SPRET/EiJ | | Replicate 1 | 176.4 | 157.1 | 125.2 (79.7%) |  |
|  |  |  | Replicate 2 | 183.1 | 164.2 | 129.7 (79.0%) |  |
|  |  |  | Replicate 3 | 157.7 | 140.4 | 110.7 (78.8%) |  |
|  | F1 hybrid | | Replicate 1 | 374.9 | 338.6 | 287.9 (85.0%) | 176.2 (61.2%) |
|  |  |  | Replicate 2 | 405.6 | 366.1 | 309.8 (84.6%) | 189.4 (61.1%) |
|  |  |  | Replicate 3 | 383.6 | 346.1 | 288.5 (83.4%) | 176.3 (61.1%) |
| Liver tissue | | C57BL/6J | Replicate 1 | 156.2 | 141.7 | 116.7 (82.4%) |  |
|  |  |  | Replicate 2 | 157.0 | 143.0 | 115.1 (80.4%) |  |
|  |  | SPRET/EiJ | Replicate 1 | 164.2 | 149.6 | 113.8 (76.1%) |  |
|  |  |  | Replicate 2 | 175.4 | 159.0 | 122.1 (76.8%) |  |
|  |  | F1 hybrid | Replicate 1 | 268.1 | 242.6 | 204.0 (84.1%) | 125.0 (61.3%) |
|  |  |  | Replicate 2 | 301.2 | 273.9 | 218.6 (79.8%) | 132.8 (60.8%) |

^1)^ The alignment rate was calculated as the number of concordantly mapped read pairs divided by the number of read pairs after trimming.

^2)^ The percentage of F1 hybrid reads which could be unambiguously assigned to allelic origin was calculated as the number of read pairs assigned to allelic origin divided by the concordantly mapped read pairs.
