# Supplementary material for: Predominant contribution of cis-regulatory divergence in the evolution of mouse alternative splicing
Source: Mol Syst Biol. 2015 Jul 1;11(7):816. doi: 10.15252/msb.20145970 (PMC4547845; doi:10.15252/msb.20145970)
Supplement: Supplementary file 3 [file msb0011-0816-sd3.doc]

Table EV2. Summary of AS events in this study

| Samples | Events | SE | RI | MXE | A3SS | A5SS | Total |
| --- | --- | --- | --- | --- | --- | --- | --- |
|  | Total number | 14,959 | 3,260 | 1,666 | 6,474 | 3,840 | 30,199 |
| Fibroblast cell line | Expressed in parental strains^1)^ | 5,615 | 1,768 | 696 | 2,236 | 1,503 | 11,818 |
|  | Divergent between parental strains^2)^ | 418 | 124 | 54 | 101 | 99 | 796 |
|  | Retained for allelic comparison^3)^ | 2,667 | 953 | 245 | 1,158 | 779 | 5,802 |
|  | Retained divergent events between  parental strains^4)^ | 203 | 69 | 21 | 63 | 61 | 417 |
|  | Divergent events in F1 hybrid^5)^ | 156 | 77 | 26 | 58 | 64 | 381 |
| Liver tissue | Expressed in parental strains^1)^ | 4,088 | 1,590 | 245 | 1,650 | 1,186 | 8,759 |
|  | Divergent between parental strains^2)^ | 286 | 143 | 18 | 84 | 76 | 607 |
|  | Retained for allelic comparison^3)^ | 1,872 | 788 | 89 | 794 | 581 | 4,124 |
|  | Retained divergent events between  parental strains^4)^ | 147 | 80 | 9 | 51 | 49 | 336 |
|  | Divergent events in F1 hybrid^5)^ | 121 | 58 | 6 | 38 | 47 | 270 |

^1)^ Number of expressed events in parental strains with at least 20 spliced-in + spliced-out supporting reads in all replicates

^2)^ Number of divergent event between parental strains at threshold BF>5 in all replicates and average |ΔPSI| >0.1.

^3)^ After filtering using mock F1 hybrid, number of expressed events in F1 hybrid with at least 20 spliced-in + spliced-out supporting reads in all replicates

^4)^ After filtering using mock F1 hybrid, number of retained divergent events between parental strains.

^5)^ Number of divergent event between the two alleles in F1 hybrid at threshold BF>5 in all replicates and average |ΔPSI| >0.1.
