# Supplementary material for: Predominant contribution of cis-regulatory divergence in the evolution of mouse alternative splicing
Source: Mol Syst Biol. 2015 Jul 1;11(7):816. doi: 10.15252/msb.20145970 (PMC4547845; doi:10.15252/msb.20145970)
Supplement: Supplementary file 6 [file msb0011-0816-sd6.pdf]

Predominant contribution of cis-regulatory divergence in the evolution of mouse alternative splicing

Mr. Qingsong Gao, Wei Sun, Marlies Ballegeer, Claude Libert and Wei Chen

Corresponding author: Wei Chen, Max-Delbrück-Centrum für Molekulare Medizin

Review timeline:

Submission date:	10 December 2014
Editorial Decision:	14 February 2015
Revision received:	13 April 2015
Editorial Decision:	20 May 2015
Accepted:	26 May 2015

Editor: Thomas Lemberger

Transaction Report:

(Note: With the exception of the correction of typographical or spelling errors that could be a source of ambiguity, letters and reports are not edited. The original formatting of letters and referee reports may not be reflected in this compilation.)

1st Editorial Decision 14 February 2015

Thank you again for submitting your work to Molecular Systems Biology and apologies for the slow process, which was due to the late arrival of the report by reviewer #3. We have now finally heard back from the three referees who agreed to evaluate your manuscript. As you will see from the reports below, the referees find the topic of your study of potential interest. They raise, however, quite substantial concerns on your work, which should be convincingly addressed in a major revision of the present study. The comments provided by the reviewers are very clear in this regards. In particular, the points raised by reviewer #2 are particularly relevant and should be carefully addressed. With regard to data quality and reproducibility of the data analysis, it is important to provide a detailed account of the methods. Do not hesitate to include scripts or literate programming tools to document your analysis pipeline.

When you resubmit your manuscript, please download our CHECKLIST (<http://msb.embopress.org/sites/default/files/additional-assets/EMBO%20Press%20Author%20Checklist%20-MSB.xlsx>) and include the completed form in your submission.

If you feel you can satisfactorily deal with these points and those listed by the referees, you may wish to submit a revised version of your manuscript. Please attach a covering letter giving details of the way in which you have handled each of the points raised by the referees. A revised manuscript will be once again subject to review and you probably understand that we can give you no guarantee at this stage that the eventual outcome will be favourable.

© European Molecular Biology Organization

REFEREE REPORTS

Reviewer #1:

Summary:

This manuscript describes a study of divergence of alternative splicing, specifically evaluating the relative contribution of cis- and trans- regulatory factors influencing splicing differences between two mouse strains. Using RNA-sequencing to quantify splicing events in the parental strains and in F1 hybrids, the authors conclude that the majority of differences are due to cis-regulatory genetic variation. The main support for this conclusion is that the majority of differences between the two strains are also present in an analysis of allele-specific splicing in F1.

Previous analyses have investigated evolution of splicing in Drosophila, and some preliminary analysis is mammals, but this is a more comprehensive analysis globally in a mammalian system, enabled by 1) the use of RNA-seq to quantify allele-specific events and 2) the use of two fairly distant mouse parental strains in order to disambiguate a large fraction of F1 reads. This study makes a useful contribution to the understanding of divergence of alternative splicing in mammals, and also provides data that may be useful for further analysis

Major points:

Allele-specific expression quantified from RNA-seq is known to be affected by significant over-dispersion (eg Skelly Genome Research 2011), meaning that in general, there are often false positives where many loci appear to display allelic imbalance simply due to technical properties of the data rather than a true biological effect. Have the authors investigated the contribution of this effect to their conclusions? In the extreme, if a very large fraction of loci display imbalance for technical reasons, even if trans effects were the dominant factor in splicing divergence, this analysis would incorrectly lead us to believe that most differences were due to cis effects. While the true contribution of over-dispersion to the reported results is almost certainly more modest than this hypothetical extreme, it realistically may contribute to a slight over-estimate of the fraction of splicing events explained by cis-effects. Since this is a major conclusion of the study, the authors should provide some analysis of this potential confounder. For example, among splicing events that *don't* differ between the two parental strains, what fraction of them appear to differ between the two alleles of the F1 hybrid? The authors should derive some estimate of the FDR, and if there appears to be any inflation of the estimate of cis-contribution, come up with a reasonable correction. If there appears to be no inflation, a supplementary figure demonstrating this should be included. (Note - the mock hybrid addresses mapping issues and other problems, but does not fully address potential over-dispersion of ASE in a real RNA-seq library.)

The analysis of genomic features that correlate with AS divergence would be more interesting if it segmented the different splicing types. Further, the analysis of potential exonic/intronic splicing enhancers is somewhat cursory (despite the minigene assay for the specific example, it's unclear what statement can be made about more general patterns) and could be expanded with some simple additional analyses - how many of each type of event were there without variants at splice sites? how many of these remaining events then have variants within known RBP binding sites, just for instance? it would be great to provide a few more basic analyses to describe the more general patterns.

Minor points:

The liver data is a nice addition to the analysis to the general conclusion that cis-effects dominate, but there are so few events that it seems questionable to draw conclusions about the specific AS sub-types.

Are the results sensitive to different choices of thresholds for statistical significance for splicing differences? Some supplementary analysis should be provided to demonstrate the high-level conclusion holds for some range of thresholds.

Presentation and style:

In the main manuscript, a brief citation or phrase to explain how "splicing site strength" was estimated, rather than just a pointer to materials/methods, would be appreciated to facilitate understanding while reading the main text.

Reviewer #2:

In the presented work, "Predominant contribution of cis-regulatory divergence in the evolution of mouse alternative splicing" the authors explore regulation of alternative splicing, in evolutionary perspective. The main goal of the paper is to estimate the relative contribution of cis versus trans regulatory events, that contribute to divergent regulation of alternative splicing in mice. To this end they employ RNAseq of two divergent strains of mice (C57BL/6 and SPRET/EiJ) and assess the cis versus trans regulation by RNAseq form F1 of these strains using allele specific expression - a hallmark of cis reguation.
I find the study design and conclusions interesting and relevant to the question. However, in my opinion, the study has some severe limitations that greatly limit the validity of authors' conclusions. I would suggest to reconsider this work for publications after the authors address the major revisions, suggested bellow. Addressing these limitations will require a significant effort on authors part, however, if they can not address them, their conclusions should be much more limited than the way it currently written.

Major concerns:
1. Data quality:
The authors use individual mouse genomes, tailored for the specific strains, which is appropriate to address the reference mapping bias, and accurate mapping of reads from the highly divergent mouse breeds used in this study. Nevertheless, authors use very permissive mapping parameters - allowing up to 10 mismatches (-N), gapped bases (--read-gap-length) and edit distance (for comparison, the default values for each of these parameters is 2). This potentially indicates an attempt to increase the number of reads mapped, to include more alternative splicing events in the analysis. Yet, despite the very permissive mapping parameters, the alignment rate is on the relatively low side (~70%) even for the parental genomes, where one would expect a much higher rate.
There are no details in materials and methods about any of the commonly used quality control procedures for preprocessing of RNAseq data (e.g. were the reads trimmed or filtered? what was the quality distribution of the data? etc...), but the combined permissive mapping parameters and relatively low mapping, suggest that the read quality might had been a problem. Alternative splicing (as opposed to total quantification of expression) is highly sensitive to the quality of aligned reads and bases, especially when mapping spliced reads that define the inferred junctions. Therefore using less than optimal quality reads may adversely affect the accuracy of alternative splicing inference, and its quantification.
In the absence of details, unfortunately I can not provide specific suggestions on the analysis parameters. Yet I would strongly encourage the authors to elaborate on quality control of their initial data and mapping, and use more stringent mapping or at least provide a comprehensive assessment how the lenient mapping parameters affect their data and conclusions.

2. Lack of power:
In this work the authors use relatively low coverage of parental strains to assess alternative splicing events (40-45M reads, also see minor comment 7 bellow). The commonly used guidelines for RNAseq sequencing depth are ~100M reads to estimate alternative splicing, and 30-50M reads to estimate differential expression (e.g. look at http://genome.ucsc.edu/ENCODE/protocols/dataStandards/ENCODE_RNAseq_Standards_V1.0.pdf). The depth of coverage and filtering parameters suggest that the current study has adequate power to examine only the relatively strong effects on alternative splicing in highly expressed genes, but probably misses the majority of the more subtle regulation or variants that have moderate or low effect sizes. Thus it is not surprising >40% of the events are a direct change in the splicing sequence, one of the probably strongest potential variant types. Based on previous studies genetic regulation of expression in cis on average has a larger effect size than the trans regulatory variants. Therefore, focusing only on the strong effects, as authors do, likely leads to gross overestimation of cis versus trans acting regulation, which does not necessarily reflect true evolutionary pressure or biological phenomena, but rather power issue. I would strongly encourage the authors to significantly increase

© European Molecular Biology Organization

the coverage for the parental strain samples.

3. Physiological relevance:
Finally the authors use RNA from cultured cells, rather than mouse tissue. It is unclear how the culturing procedure affects alternative splicing, but in the last section of the results the authors describe examining AS pattern of 55 events of interest in previously published data from brain tissue, and only 6 of them (~11%) seem to correspond between the fibroblasts and the brain. I find 11% to be a troublingly low replication rate and suggest that the authors explore this issue further. Given that there are abundant high coverage RNAseq datasets that were published on various tissues in C57BL/6 and other strains, which are available either through European sequence archive or through the NCBI GEO database, I would suggest the authors make a serious effort to asses how well the AS patterns in their data correspond to the ones observed in actual mouse tissues. If indeed the fibroblasts cultures do not reflect the commonly observed picture in mouse tissues, this would suggest that the conclusions of this study do not necessarily pertain to evolutionary process, but may reflect some unknown effect of the culturing procedure that may emphasize cis regulation over trans.

Minor comments
1. I find the article well written and organized, however the clarity of the presentation and the amount of detail greatly decreases along the manuscript progression. I would suggest the authors pay more attention to details along the entire text.
2. Given the major comments above, I find the author's conclusions overstated and would suggest a more careful discussion of potential limitations of their dataset.
3. Figure legends lack detail, for example the authors fail to explain all the symbols in figure 2B and E3 C (what are the asterisks? what are the black and grey colors?). There are no table legends, table E3 is designated as S3, and table E4 seems to be missing. The figures are not clearly labeled, and while I was able to deduce which is which, I would suggest the authors pay more attention to such details.
4. In addition to minor point 3, from visual observation it seems figure 2B and E3 C are actually plotting of the same exact data with different designation of cis and trans. This is an unclear point, that should be clearly explained in the manuscript to avoid confusion.
5. Figure E3: panels A and B suggest that there 773 divergent AS events between the parental strains, and 376 in the F1s. In panel C however, they refer to 405 of these events. The mismatch between the numbers is completely unclear, and is not explained in the main text or the figure legend.
6. As mentioned in major point 1, material and methods have absolutely no information on the preprocessing and quality control procedures undertaken to avoid mapping low quality bases and reads. I would suggest the authors provide more detail on the mapping procedure.
7. Read counts and table E1 - it is unclear whether the counts refer to pairs of reads or to individual reads. This is an important issue in assessing the adequacy of depth of coverage used in this study (please also see major comment 2).

Reviewer #3:

Summary
The authors use RNA-seq data from two mouse strains (C57BL/6J, SPRET/EiJ) as well as the resulting F1 hybrid, along with already published data to study the influence of cis and trans-regulatory variants on alternative splicing in mammals. Using RNA-seq data derived from mouse fibroblast cell lines, the authors are able to identify 382 and 219 differentially regulated splicing events between the two mouse strains and between the two alleles in the F1 hybrid respectively. The authors found that the distribution of the five types of splicing events studied is relatively uniform with the exception of A3SS. Next, analyzing the F1 hybrid AS events, the authors find 234 events that had divergent regulation in the parental strains of which 140 shown cis- and 38 and trans-divergence. To test the whether the predominant cis-contribution to the AS is uniform across other mouse strains, the authors analyze AS patterns between the C57BL/6J and CAST/EiJ strains previously published data. The results show a dominant contribution of cis-regulatory divergence for all five types of AS events. Finally the authors investigate the distribution genomic features that correlated with cis-reuglatory AS divergence. The authors found that variants changing the canonical GU/AG splicing sites severely affect the splicing site strength. The authors also find a

differential contributions of cis-regulatory variants beyond the canonical splicing site. Using PacBio sequencing and RT-PCR, the authors were able to validate a sample of 20 AS events in both the parental strain and F1 hybrid. The authors use a variety of statistical tests (Bayesian factor, Fisher'e exact test) to ensure the accuracy of their analysis and to eliminate any test dependent bias.

Good points
· The study supports the hypothesis that cis-regulatory variants are the dominant contributors to the AS divergence in mouse strains. And combining these results with previous similar results between human and mouse, and human and chimp, it is possible to say that this hypothesis holds true for mammals in general.
· Using F1 hybridng the authors are able to identify unambiguously cis-regulatory divergence allele-specific splicing patterns, since the nascent RNA transcripts form both parental alleles are subject to the same trans-regulatory environments.
· Good statistical analysis - the authors make use of previously defined Bayesian factor thresholds for estimating the statistical significance of the splicing difference.
· Use of two strains divergent over 1.5 MY similar to Drosophila
- for comparison purpose.

Points that can use improvement :
· why does the percentage of differential splicing events converage of the A3SS is lower (2.8%) compared to the coverage of all the other events (~4.5%)
· minor point: à in the "Predominant contribution of cis-regulatory variants..." section, they say only 30% of the F1 (C57BL/6J-CAST/EiJ) reads were mapped unambiguously compared to their original F1 hybrid where they were able to map 60 % of the reads , however earlier in the section they say that actually only 72.8 out of 170 million reads were mapped unambiguously, and this actually makes for 40%, (not 60%)
· Out of the 234 F1 AS events that contain 140 cis and 38 trans divergence, what happens with the rest of 56 are they neither cis nor trans?
· Also since only 219 F1 AS events are above the threshold for being divergent using BF >5 and dPSI > 0.1, what proportion of the 234 events are actually divergent by passing the test?
· The novelty of the study can be questionable since a dominant cis-contribution to the AS in mammals has been observed before in comparative studies in human& mouse (Barbosa-Morais 2012) and human& chimp (Lin 2010), but this study reinforces the generality of the hypothesis over the mouse strain
· While the discussion of the AS in fly highlights the differences between the two studies it does not give a satisfactory explanation of the dominance of trans-effects in the exon skipping events in fly and merely states the obvious, so I believe that the fly part does not add anything to the conclusion of the paper.

1st Revision - authors' response 13 April 2015

© European Molecular Biology Organization

Detailed point-by-point response

Reviewer 1

Summary:

This manuscript describes a study of divergence of alternative splicing, specifically evaluating the relative contribution of cis- and trans- regulatory factors influencing splicing differences between two mouse strains. Using RNA-sequencing to quantify splicing events in the parental strains and in F1 hybrids, the authors conclude that the majority of differences are due to cis-regulatory genetic variation. The main support for this conclusion is that the majority of differences between the two strains are also present in an analysis of allele-specific splicing in F1.

Previous analyses have investigated evolution of splicing in Drosophila, and some preliminary analysis is mammals, but this is a more comprehensive analysis globally in a mammalian system, enabled by 1) the use of RNA-seq to quantify allele-specific events and 2) the use of two fairly distant mouse parental strains in order to disambiguate a large fraction of F1 reads. This study makes a useful contribution to the understanding of divergence of alternative splicing in mammals, and also provides data that may be useful for further analysis

Major points

1. Allele-specific expression quantified from RNA-seq is known to be affected by significant over-dispersion (e.g. Skelly Genome Research 2011), meaning that in general, there are often false positives where many loci appear to display allelic imbalance simply due to technical properties of the data rather than a true

biological effect. Have the authors investigated the contribution of this effect to their conclusions? In the extreme, if a very large fraction of loci display imbalance for technical reasons, even if trans effects were the dominant factor in splicing divergence, this analysis would incorrectly lead us to believe that most differences were due to cis effects. While the true contribution of over-dispersion to the reported results is almost certainly more modest than this hypothetical extreme, it realistically may contribute to a slight over-estimate of the fraction of splicing events explained by cis-effects. Since this is a major conclusion of the study, the authors should provide some analysis of this potential confounder. For example, among splicing events that *don't* differ between the two parental strains, what fraction of them appear to differ between the two alleles of the F1 hybrid? The authors should derive some estimate of the FDR, and if there appears to be any inflation of the estimate of cis-contribution, come up with a reasonable correction. If there appears to be no inflation, a supplementary figure demonstrating this should be included. (Note - the mock hybrid addresses mapping issues and other problems, but does not fully address potential over-dispersion of ASE in a real RNA-seq library.)

A: We thank the referee for pointing out the potential overestimation of allelic AS divergence and his/her important suggestion on FDR estimation. Following his/her suggestion,

1) We formally calculated the FDR based on the three biological replicate data, using an approach previously described by Sterne-Weiler et al ((Sterne-Weiler et al., 2013), please see Materials and methods for details). In brief, for each value of x from 0.01 to 0.20 increasing by 0.01, we performed independent 100 bootstrapped label permutations of other replicates. For each of the 100 shuffled sets, we calculated the

number of events passing the threshold (false positives), *i.e.* BF > 5 in all the replicates and average |ΔPSI| >x. Then for each of the 100 permutations of each value x, the FDR was estimated as false positives divided by the number of real events passing the threshold, including both false positives and true positives.

As shown in Figure E1, at the threshold we chose (BF>5 in all the three replicates and average |ΔPSI|>0.1), the FDR of parental strains and F1 hybrid are nearly identical (2.5% vs 2.4%), indicating that compared to parental divergency, allelic divergency is not over-estimated.

2) We further checked the data dispersion for both parental strains and F1 hybrid. A similar sliding window approach as described in "Filter with mock F1 hybrid" section of Materials and methods was used to calculate the standard deviation for each of the 50 windows (2% of total events in each window). As shown in the MA plots comparing the estimated ΔPSI values between two replicates data from parental strains (Fig R1 A) or between two replicates from F1 hybrid (Figure R1 B), the dispersions were clearly dependent on the number of event-supporting sequencing reads, for both parental and allelic comparisons. As expected, the higher the expression level, the lower the noise. Importantly, there was no difference in the dispersion, *i.e.* local standard deviation of ΔΔPSI in each of the 50 windows, between parental strains and F1 hybrid (Figure R1 C), again arguing against the potential overestimation of allelic versus parental AS divergence.

A

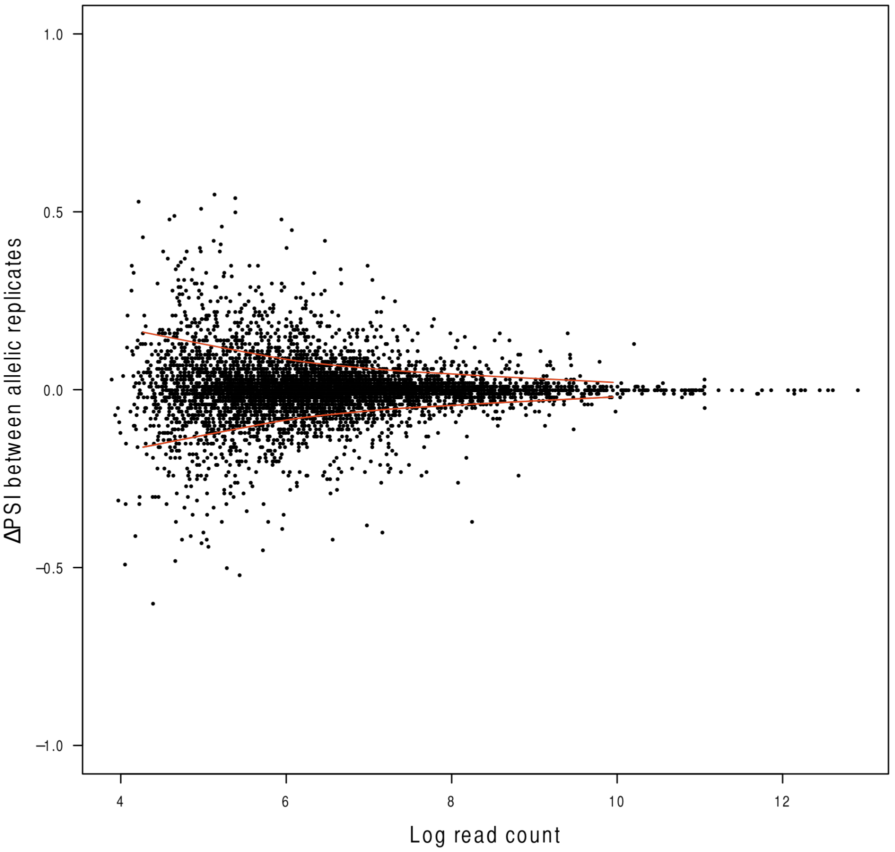
ΔPSI between allelic replicates
1.0
0.5
0.0
−0.5
−1.0
4
6
8
10
12
Log read count

B

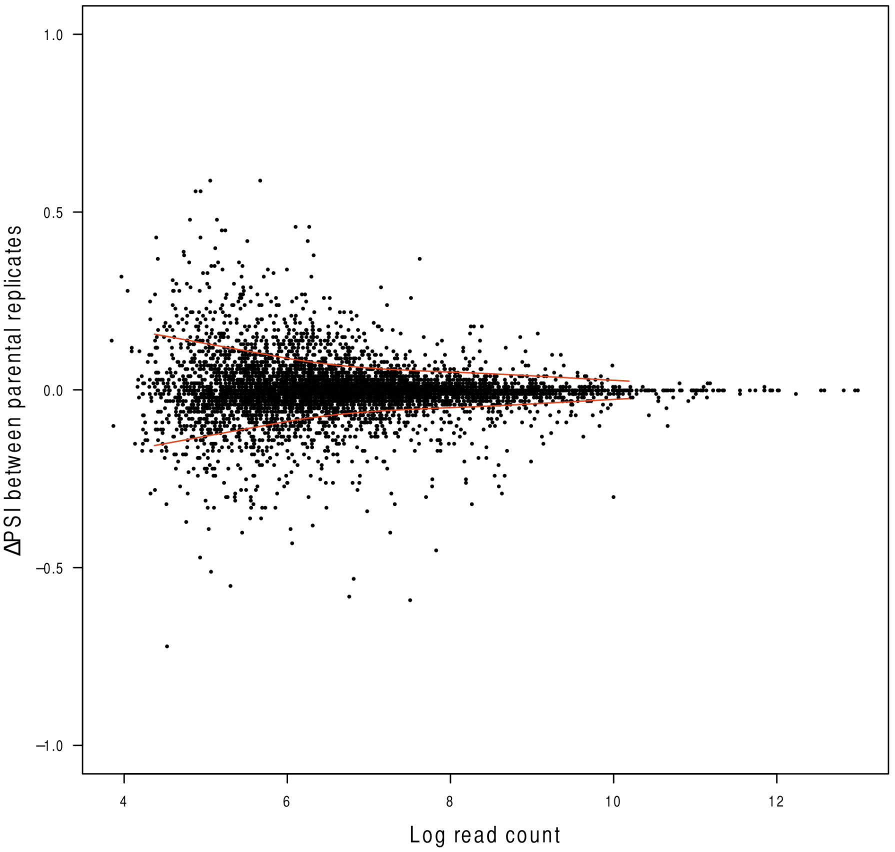
ΔPSI between parental replicates
1.0
0.5
0.0
−0.5
−1.0
4
6
8
10
12
Log read count

C

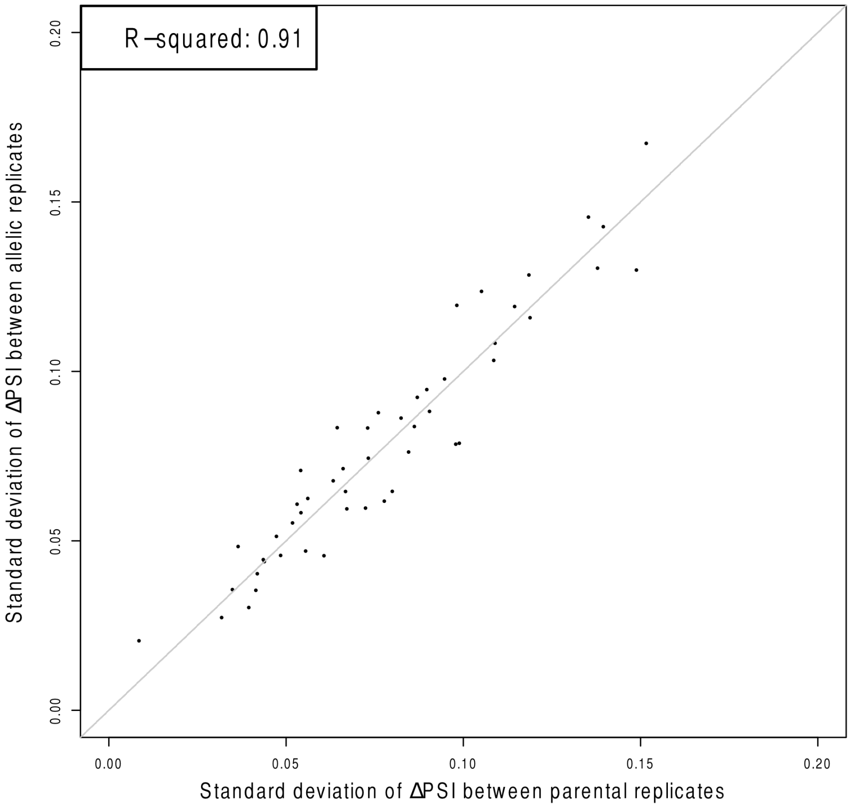

Figure R1. (A) MA plot comparing allelic ΔPSI values estimated between the two replicates of F1 hybrid from parental strains. The log transformed read count was plotted in x axis, and ΔPSI difference (ΔΔPSI) was plotted in y axis. The local standard deviation was also indicated in red lines. (B) MA plot comparing parental ΔPSI values between the two replicates from parental strains (C) The dispersion of ΔPSI values, *i.e.* local standard deviation of ΔΔPSI in each of the 50 windows, correlated well between parental strains (x axis) and F1 hybrid (y axis) (R^2=0.91).

3) To assess the accuracy of our AS analysis based on Illumina approach, especially for allelic analysis, we selected 20 candidate events consisting of all five different AS types (8 SE, 3 RI, 3 MXE, 2 A3SS, and 4 A5SS) for validation using PacBio RS system (see main text). As shown in Fig 2A, the splicing changes estimated in this way were significantly correlated with those determined by Illumina RNA-seq, for both parental and allelic comparison (R^2=0.91 and 0.92 for parental and allelic

comparison, respectively).

All our data and analysis elaborated above support that we did not overestimate allelic versus parental AS divergence, therefore our conclusion on predominant *cis*-contribution could not be due to technically inflated estimation of allelic AS divergence. Please note that "splicing events that don't differ between the two parental strains, but differ between the two alleles in F1 hybrid" could be due to the compensatory effect between *cis*- and *trans*- divergence, and therefore should not be used to represent false positives.

2. The analysis of genomic features that correlate with AS divergence would be more interesting if it segmented the different splicing types. Further, the analysis of potential exonic/intronic splicing enhancers is somewhat cursory (despite the minigene assay for the specific example, it's unclear what statement can be made about more general patterns) and could be expanded with some simple additional analyses - how many of each type of event were there without variants at splice sites? how many of these remaining events then have variants within known RBP binding sites, just for instance? it would be great to provide a few more basic analyses to describe the more general patterns.

A: We have now re-analyzed the genomic features that correlate with AS divergence for each splicing type and summarized the results in the Figure E9, which includes the percentage of nucleotide variants and the percentage of events with/without nucleotide difference on splice sites.

We also analyzed the global effect of potential exonic/intronic splicing enhancer/silencers, as the referee suggested. For this purpose, we downloaded all 294 AS regulatory sequence motifs from RegRNA 2.0 web server

(http://regrna2.mbc.nctu.edu.tw/) (Chang et al., 2013)), and retained the 79 motifs of length 6~10 nucleotides annotated for human, mouse or rat (including 44 exonic splicing enhancers, 10 exonic splicing silencers, 15 intronic splicing enhancers and 10 intronic splicing silencers). To compare with the *cis*-regulatory divergent events, we selected a set of the control events with similar overall nucleotide diversity in the flanking regions (see Fig R2A). We then scanned the flanking regions (exons and introns, separately) of each AS event to count the number of splicing motifs affected by nucleotide variants. Finally we compared it between the events with *cis*-regulatory divergence and control events. In the exonic regions, only 22 (out of 243) divergent events have at least one motif-affecting sequence variants, compared to 33 (out of 283) control events. In the intronic regions, only 58 (out of 243) divergent events have at least one motif-affecting sequence variants, compared to 62 (out of 283) control events. As shown in Figure R2 B-C, there is no significant difference between the divergent and control events. Although this could be due to the lack of statistical power, very likely, this observation also reflects our limited understanding of 'splicing code' in mammals.

A

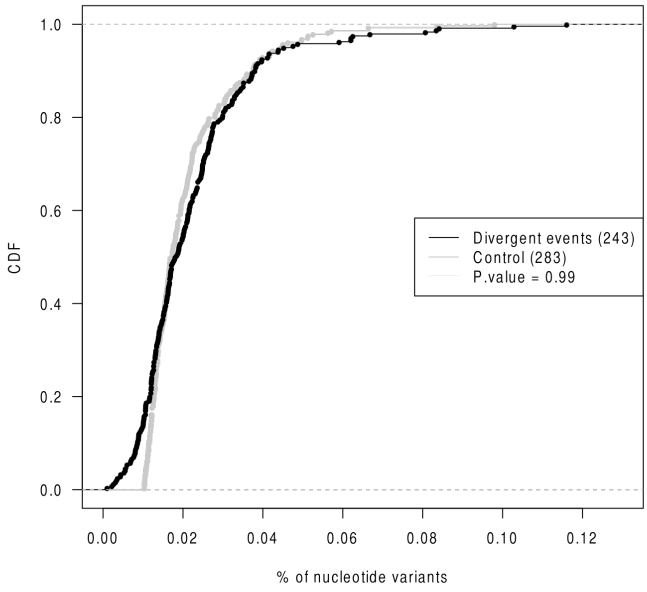

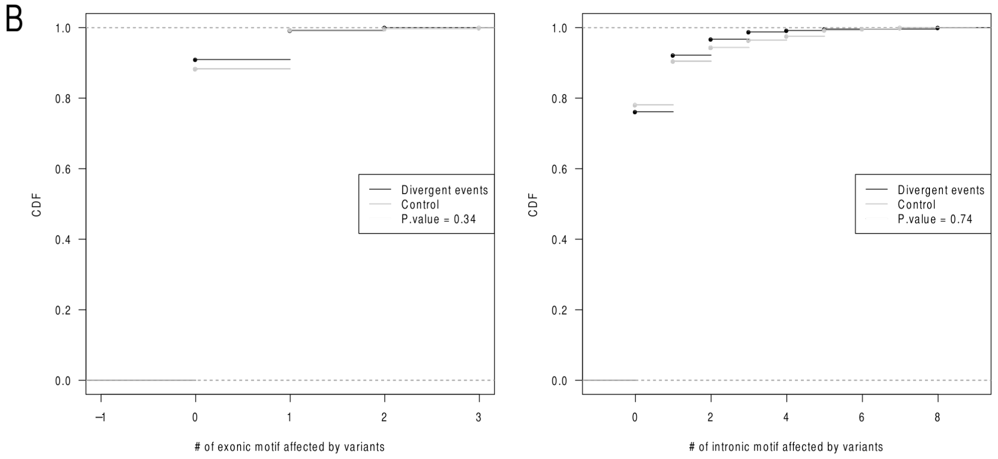

Fig R2. (A) CDF of number of nucleotide variants in the AS flanking regions for the events with *cis*-regulatory divergence (black) and controls (gray), which was chosen with similar overall nucleotide diversity. (B) CDF of number of splicing regulatory motifs in the flanking exonic (left) or intronic (right) regions affected by sequence variants beyond splice sites.

Minor points

1. The liver data is a nice addition to the analysis to the general conclusion that cis-effects dominate, but there are so few events that it seems questionable to draw conclusions about the specific AS sub-types.

A: To check whether our conclusion from cultured cells could be extended to mouse tissues, in the revised manuscript, we performed RNA-seq for two replicates of the liver samples from C57BL/6J, SPRET and their F1 hybrid, respectively, at a similar sequencing depth as achieved now for fibroblast cells. As shown in Figure E6, in liver samples, we observed again a predominant contribution of *cis*-regulatory divergence consistently across all the five AS types (please see main text for more detail).

2. Are the results sensitive to different choices of thresholds for statistical

significance for splicing differences? Some supplementary analysis should be provided to demonstrate the high-level conclusion holds for some range of thresholds.

A: To check whether our conclusion is sensitive to difference thresholds, we tried different cutoffs of |ΔPSI| values corresponding to different FDRs (see Figure E1). As shown in the Figure E4A-C, *cis*-regulatory divergence always showed predominant contributions at different thresholds (|ΔPSI|>0.0, 0.05 and 0.15, respectively). This trend held true for all the five AS types (Fig E4D-F).

In addition, we also checked whether the *cis*-/*trans*- contributions were different for parental divergent events with different effect sizes (*i.e.* ΔPSI). For this, we grouped the 417 divergent events between parental strains into 7 categories according to the |ΔPSI| values: (0.1, 0.2], (0.2, 0.3], (0.3, 0.4], (0.4, 0.5], (0.5, 0.6], (0.6, 0.7] and (0.7, 1.0]. As shown in Figure E4G, while *cis*-regulatory divergence always played a predominant role in determining parental AS divergence with different effect sizes, its relative contribution slightly decreased with the decreasing of effect sizes.

3. In the main manuscript, a brief citation or phrase to explain how "splicing site strength" was estimated, rather than just a pointer to materials/methods, would be appreciated to facilitate understanding while reading the main text.

A: We have now added the following details about splicing site strength into the main text: "Sequence variants at splice sites could regulate alternative splicing by affecting splice site strength – the probability that the splice sites could be recognized by the spliceosome (McManus et al., 2014). To investigate how sequence variants at the splicing sites could affect splicing site strength, we calculated the splicing site strength score for the two alleles containing variants at the exact splice sites (Material

and methods) and compared the allelic difference of such score between the events with *cis*-regulatory divergence and those without. As shown in Fig 3C, the sequence variants at the splicing sites of *cis*-divergent events affected the splicing site strength more than those at splicing sites of control events. ”

Reviewer 2:

In the presented work, "Predominant contribution of cis-regulatory divergence in the evolution of mouse alternative splicing" the authors explore regulation of alternative splicing, in evolutionary perspective. The main goal of the paper is to estimate the relative contribution of cis versus trans regulatory events, that contribute to divergent regulation of alternative splicing in mice. To this end they employ RNAseq of two divergent strains of mice (C57BL/6 and SPRET/EiJ) and assess the cis versus trans regulation by RNAseq form F1 of these strains using allele specific expression - a hallmark of cis regulation.

I find the study design and conclusions interesting and relevant to the question. However, in my opinion, the study has some severe limitations that greatly limit the validity of authors' conclusions.

I would suggest to reconsider this work for publications after the authors address the major revisions, suggested bellow. Addressing these limitations will require a significant effort on authors part, however, if they can not address them, their conclusions should be much more limited than the way it currently written.

Major points

1. Data quality:

The authors use individual mouse genomes, tailored for the specific strains,

which is appropriate to address the reference mapping bias, and accurate mapping of reads from the highly divergent mouse breeds used in this study. Nevertheless, authors use very permissive mapping parameters - allowing up to 10 mismatches (-N), gapped bases (--read-gap-length) and edit distance (for comparison, the default values for each of these parameters is 2). This potentially indicates an attempt to increase the number of reads mapped, to include more alternative splicing events in the analysis. Yet, despite the very permissive mapping parameters, the alignment rate is on the relatively low side (~70%) even for the parental genomes, where one would expect a much higher rate. There are no details in materials and methods about any of the commonly used quality control procedures for preprocessing of RNAseq data (e.g. were the reads trimmed or filtered? what was the quality distribution of the data? etc...), but the combined permissive mapping parameters and relatively low mapping, suggest that the read quality might had been a problem. Alternative splicing (as opposed to total quantification of expression) is highly sensitive to the quality of aligned reads and bases, especially when mapping spliced reads that define the inferred junctions. Therefore using less than optimal quality reads may adversely affect the accuracy of alternative splicing inference, and its quantification. In the absence of details, unfortunately I can not provide specific suggestions on the analysis parameters. Yet I would strongly encourage the authors to elaborate on quality control of their initial data and mapping, and use more stringent mapping or at least provide a comprehensive assessment how the lenient mapping parameters affect their data and conclusions.

A: Sorry for not having provided the details on our mapping procedure. Following the suggestions, we changed the mapping parameters to the default ones and added more

detail information to the revised Materials and methods, including:

1) Preprocessing of RNA-seq reads

Flexbar was first used to trim the RNA-seq reads that pass the Illumina filter to remove library adapter sequences with parameters -f i1.8 -x 6 -u 0 -m 90 -k 90 -ae RIGHT (Dodt et al., 2012). Here in addition to the adapter sequences, we trimmed the first 6 bases on 5' end to remove the sequence artifact due to the use of random hexamer as RT primers (-x 6). We retained only the read pairs with both reads of length >= 90 nucleotides after adapter removal (-m 90) and trimmed all of them from 3'end to the same length of 90 nucleotides (-k 90).

2) RNA-seq read mapping

Following the referee's suggestion, we used the default parameters of TopHat for mapping the RNA-seq reads.

Using such setting, on average 86% and 79% of the remaining read pairs from C57BL/6J and SPRET/EiJ strains could be mapped to the corresponding genome references, respectively. We have added one more column to Table E1 and defined the percentage of mappable read pairs in more details.

To clarify the low mapping rate as pointed out by the referee, the previous "70%" was calculated by dividing the number of reads mapped to autosome by the number of total sequencing reads before trimming. The alignment rate was more than 80% for both strains (permissive mapping parameters before) if we divided the number of reads mapped to all the chromosomes by the total number of reads after trimming.

3) RNA-seq quality control

To assure the high quality of our sequencing reads, we used read_quality.py script from RSeQC package to check the base calling quality (Wang et al., 2012). As shown

in Figure R3, the Phred quality scores for all the remaining 90 bases were excellent for both parental strains and F1 hybrid.

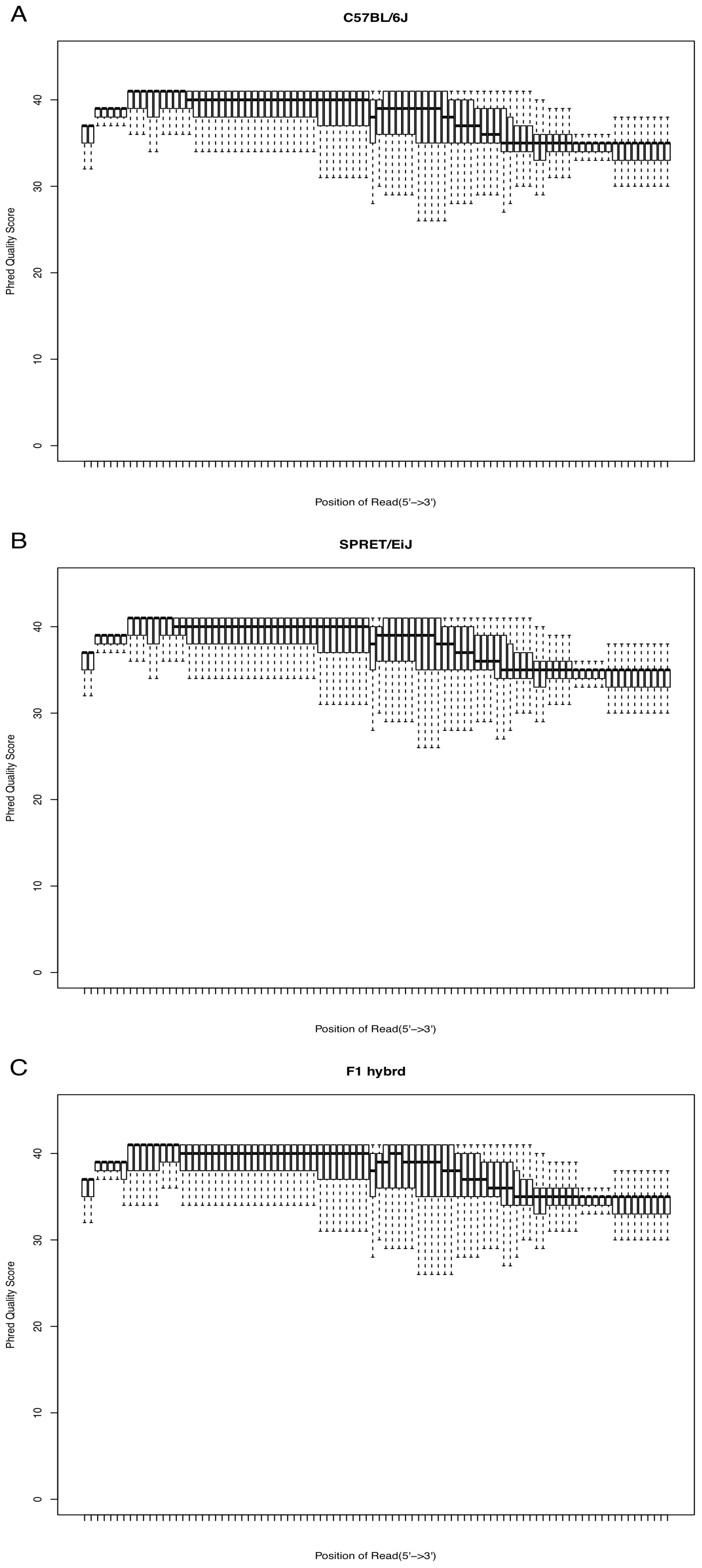

Fig R3. The distribution of Phred quality scores for all the remaining 90 bases in the RNA-seq

dataset from C57BL/6J (A), SPRET/EiJ (B) and their F1 hybrid (C).

2. Lack of power:

In this work the authors use relatively low coverage of parental strains to assess alternative splicing events (40-45M reads, also see minor comment 7 bellow). The commonly used guidelines for RNAseq sequencing depth are ~100M reads to estimate alternative splicing, and 30-50M reads to estimate differential expression (e.g. look at http://genome.ucsc.edu/ENCODE/protocols/dataStandards/ENCODE_RNAseq_Standards_V1.0.pdf). The depth of coverage and filtering parameters suggest that the current study has adequate power to examine only the relatively strong effects on alternative splicing in highly expressed genes, but probably misses the majority of the more subtle regulation or variants that have moderate or low effect sizes. Thus it is not surprising >40% of the events are a direct change in the splicing sequence, one of the probably strongest potential variant types. Based on previous studies genetic regulation of expression in cis on average has a larger effect size than the trans regulatory variants. Therefore, focusing only on the strong effects, as authors do, likely leads to gross overestimation of cis versus trans acting regulation, which does not necessarily reflect true evolutionary pressure or biological phenomena, but rather power issue. I would strongly encourage the authors to significantly increase the coverage for the parental strain samples.

A: We thank the referee for this critical comment. Following the suggestion, we re-sequenced all the 9 fibroblast samples (six from parental lines and three from F1 hybrid), and boosted the sequencing depth to on average 169.4 and 388.0 million read pairs for parental and F1 samples (see Table E1). Please note in F1 samples, the

number of reads that could be assigned to allelic origin is similar as that from parental samples, therefore we have a similar detection power for both parental and *cis*-divergent events (Table E1). As shown in Fig E1, the FDR rate is also nearly identical for detecting parental versus allelic AS divergent event.

Based on the down-sampling analysis, in which we randomly selected a subset of sequencing data with different no. read pairs (from 20 million to 160 million increasing by 20 million for parental strains, and from 40 million to 360 million increasing by 40 million for F1 hybrid), the number of expressed AS events approached saturation at current sequencing depth (Figure R4). With current sequencing depth, we in total identified 796 divergent AS events between parental strains (382 in previous version).

We also checked whether the *cis-/trans-* contributions were different for parental divergent events with different effect sizes (*i.e.* ΔPSI). For this, we grouped the 417 divergent events between parental strains into 7 categories according to the |ΔPSI| values: (0.1, 0.2], (0.2, 0.3], (0.3, 0.4], (0.4, 0.5], (0.5, 0.6], (0.6, 0.7] and (0.7, 1.0]. As shown in Figure E4G while *cis*-regulatory divergence always played a predominant role in determining parental AS divergence with different effect sizes, its relative contribution slightly decreased with the decreasing of effect sizes. We added this part into the revised manuscript. Please note Figure R5 below showed that this trend held true even for much smaller effect size |ΔPSI|∈(0.0, 0.1].

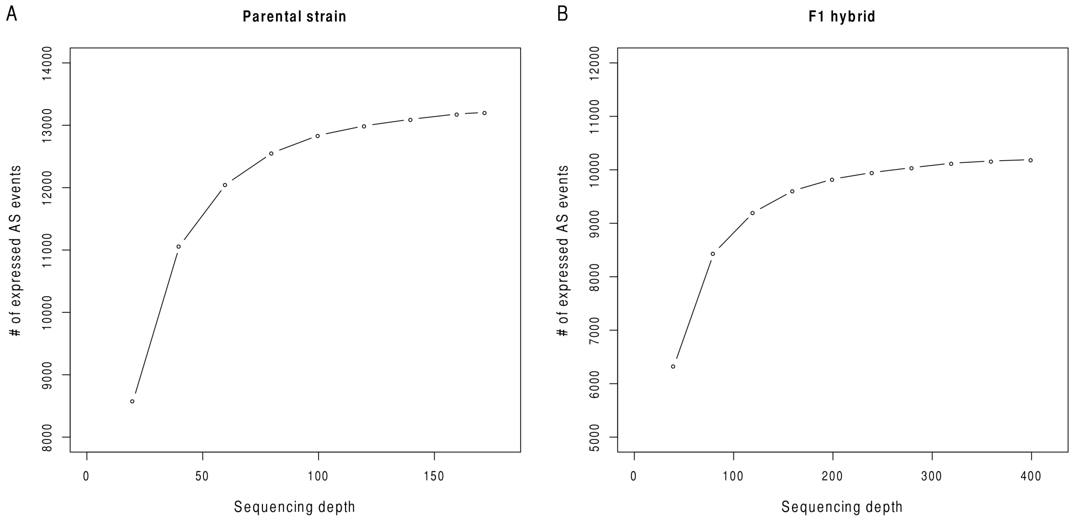

Fig R4. The number of expressed AS events (y-xis) was plotted against different down-sampling sequencing depth (x-axis) for parental strains (A) and F1 hybrid (B).

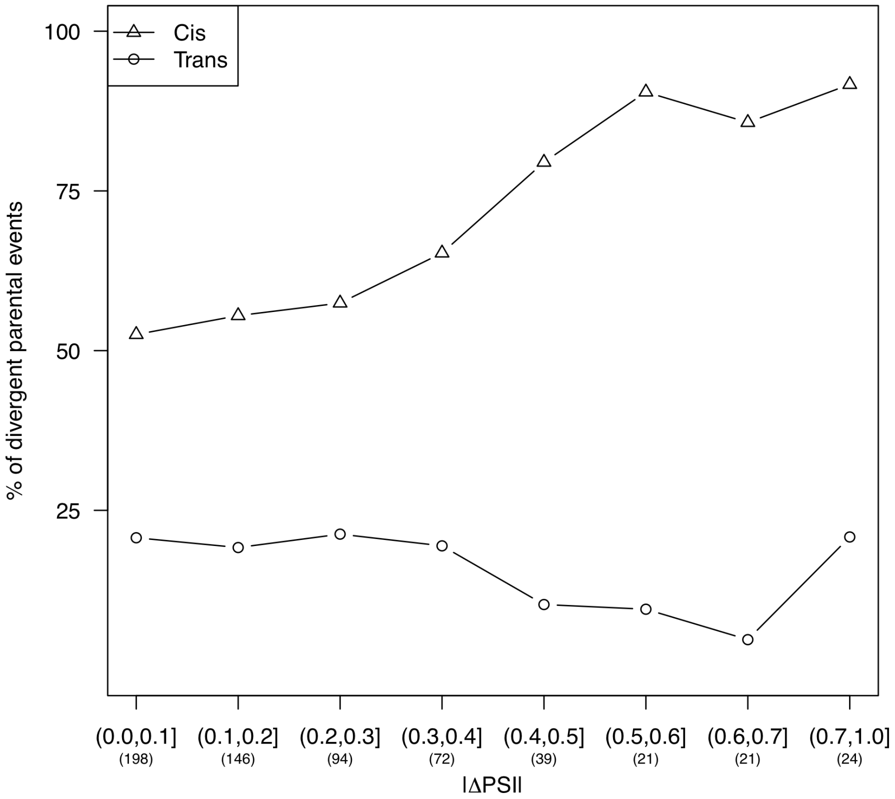

Fig R5. Contributions of *cis* (indicated as triangle)-/*trans* (indicated as circle)- regulatory divergence (y-axis) for parental divergent splicing events with different effect sizes (*i.e.* |ΔPSI|,

x-axis). 615 divergent events between parental strains for |ΔPSI|>0 (see Figure E4A) were grouped into 8 categories according to the |ΔPSI| values: (0, 0.1], (0.1, 0.2], (0.2, 0.3], (0.3, 0.4], (0.4, 0.5], (0.5, 0.6], (0.6, 0.7] and (0.7, 1.0]. The number of events in each category was marked accordingly.

3. Physiological relevance:

Finally the authors use RNA from cultured cells, rather than mouse tissue. It is unclear how the culturing procedure affects alternative splicing, but in the last section of the results the authors describe examining AS pattern of 55 events of interest in previously published data from brain tissue, and only 6 of them (~11%) seem to correspond between the fibroblasts and the brain. I find 11% to be a troublingly low replication rate and suggest that the authors explore this issue further. Given that there are abundant high coverage RNAseq datasets that were published on various tissues in C57BL/6 and other strains, which are available either through European sequence archive or through the NCBI GEO database, I would suggest the authors make a serious effort to asses how well the AS patterns in their data correspond to the ones observed in actual mouse tissues. If indeed the fibroblasts cultures do not reflect the commonly observed picture in mouse tissues, this would suggest that the conclusions of this study do not necessarily pertain to evolutionary process, but may reflect some unknown effect of the culturing procedure that may emphasize cis regulation over trans.

A: We thank the referee again for the important comment. To check whether our conclusion previously based on cultured fibroblast cells could be generalized to mouse tissues, in addition to the re-analysis of published datasets with much lower sequencing depth, we sequenced two replicates of the liver samples from C57BL/6J, SPRET and their F1 hybrid, with a sequencing depth of on average 163.2 and 284.6

million read pairs for parental and F1 samples (Table E1).

Out of 8,759 AS events expressed in the parental samples, 607 were identified as significantly divergent between the parental strains (BF>5 in both replicates and average |ΔPSI|>0.1). After the similar filtering based on mock F1 dataset, 4,124 and 336 total expressed and divergent events retained, respectively (Table E2). Then by applying the same threshold as that for parental strains, we detected 270 divergent events between the two alleles in F1 hybrid (Table E2). Finally we compared the allelic divergent to the parental divergent AS. Out of 336 parental divergent events retained after filtering, 196 and 38 exhibited significant *cis*- and *trans*-regulatory divergence, respectively. Such predominant contributions of *cis*-regulatory divergence were also evident for all the five splicing types (Fig E6B). We added this part into the revised manuscript.

The lower overlap of divergent AS between our fibroblast data and published brain data was largely due to the lower sequencing depth of published brain data. Based on our own sequencing data from fibroblast and liver samples, as shown in Figure R6, 597 and 234 of the parental and allelic divergent events identified in fibroblast cell line were also expressed in liver tissues, of which 227 (38%) and 121 (52%) could be identified also as parental and allelic divergent in liver tissues, respectively.

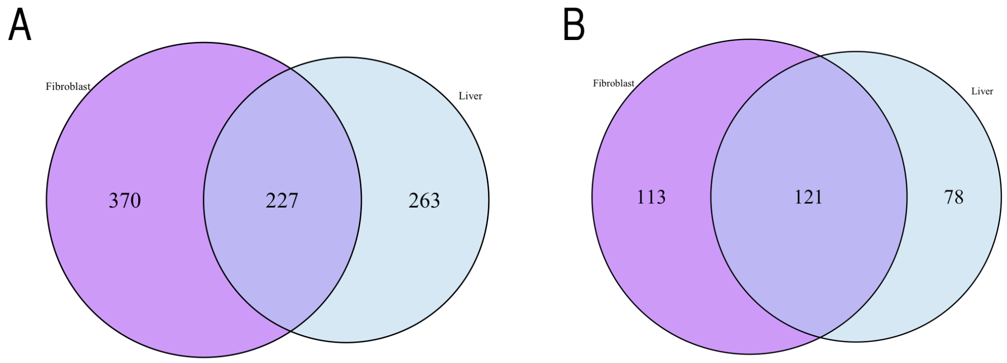

Fig R6. Venn diagram showing the overlap of the divergent events identified from fibroblast cell line and liver in parental strains (A) and in F1 hybrid (B). Only the events with at least 20 supporting reads in both fibroblast cell line and liver were used for the comparison.

Minor points

1. I find the article well written and organized, however the clarity of the presentation and the amount of detail greatly decreases along the manuscript progression. I would suggest the authors pay more attention to details along the entire text.

A: In the revised manuscript, we have now added more details to our main text, Materials and method, figure legends and etc.

2. Given the major comments above, I find the author's conclusions overstated and would suggest a more careful discussion of potential limitations of their dataset.

A: As explained in the response to main points, in the revision, we 1) adjusted mapping parameters to assure good mapping quality; 2) increased sequencing depth to assure almost saturated detection sensitivity; 3) added our own RNA-seq data from

liver tissues at very high sequencing depth. We believed that these measures should address the concerns of the referee.

3. Figure legends lack detail, for example the authors fail to explain all the symbols in figure 2B and E3 C (what are the asterisks? what are the black and grey colors?). There are no table legends, table E3 is designated as S3, and table E4 seems to be missing. The figures are not clearly labeled, and while I was able to deduce which is which, I would suggest the authors pay more attention to such details.

A: We have added more detailed description to the figure legends and table legends

Grey dots in Figure 2B represented 5,802 AS events expressed in F1 hybrid after filtering using mock F1 hybrid.

"+" and "x" were used to indicate significant *cis*- and *trans*-regulatory divergence, respectively. Therefore, asterisks represent the events with both significant *cis*- and *trans*-regulatory divergence.

We carefully checked and corrected the mis-label of all supplementary tables. Table E4 could be opened properly in the MSB online system. Please note that there were two sheets in Table E4.

4. In addition to minor point 3, from visual observation it seems figure 2B and E3 C are actually plotting of the same exact data with different designation of cis and trans. This is an unclear point that should be clearly explained in the manuscript to avoid confusion.

A: Indeed, Figure 2B and Figure E3C plotted the same data, but different designation of *cis*- and *trans*- events based on MISO and Fisher's exact test methods, respectively. We would like to demonstrate that our conclusion would not be affected by using

specific statistical methods. We made it clear in the revised manuscript.

5. Figure E3: panels A and B suggest that there 773 divergent AS events between the parental strains, and 376 in the F1s. In panel C however, they refer to 405 of these events. The mismatch between the numbers is completely unclear, and is not explained in the main text or the figure legend.

A: In the previous Figure E3 (now E5), panels A and B shows the overlap of the divergent events identified by Fisher's exact test and MISO in parental strains (A) and F1 hybrid (B). The total numbers of divergent events between parental strains are 765 and 382 for Fisher's exact test and MISO, respectively. And the total numbers of divergent events between the two alleles are 374 and 219 for Fisher's exact test and MISO, respectively. In Panel C, the number of parental divergent events (based on Fisher's exact test) represented those remained after mocking filtering. We have added more detailed explanation in the revised figure legends.

6. As mentioned in major point 1, material and methods have absolutely no information on the preprocessing and quality control procedures undertaken to avoid mapping low quality bases and reads. I would suggest the authors provide more detail on the mapping procedure.

A: We added more details about preprocessing and quality control procedure to the Materials and methods.

7. Read counts and table E1 - it is unclear whether the counts refer to pairs of reads or to individual reads. This is an important issue in assessing the adequacy of depth of coverage used in this study (please also see major comment 2).

A: All the read counts in the manuscript refer to read pairs. We clarified this in the revised manuscript.

Reviewer 3:

Summary

The authors use RNA-seq data from two mouse strains (C57BL/6J, SPRET/EiJ) as well as the resulting F1 hybrid, along with already published data to study the influence of *cis* and *trans*-regulatory variants on alternative splicing in mammals. Using RNA-seq data derived from mouse fibroblast cell lines, the authors are able to identify 382 and 219 differentially regulated splicing events between the two mouse strains and between the two alleles in the F1 hybrid respectively. The authors found that the distribution of the five types of splicing events studied is relatively uniform with the exception of A3SS. Next, analyzing the F1 hybrid AS events, the authors find 234 events that had divergent regulation in the parental strains of which 140 shown cis- and 38 and trans-divergence. To test the whether the predominant cis-contribution to the AS is uniform across other mouse strains, the authors analyze AS patterns between the C57BL/6J and CAST/EiJ strains previously published data. The results show a dominant contribution of cis-regulatory divergence for all five types of AS events. Finally the authors investigate the distribution genomic features that correlated with cis-reuglatory AS divergence. The authors found that variants changing the canonical GU/AG splicing sites severely affect the splicing site strength. The authors also find a differential contributions of cis-regulatory variants beyond the canonical splicing site. Using PacBio sequencing and RT-PCR, the authors were able to validate a sample of 20 AS events in both the parental strain and F1 hybrid. The authors use a variety of statistical tests (Bayesian factor, Fisher'e exact test) to ensure the accuracy of their analysis and to eliminate any test dependent bias.

Good points

The study supports the hypothesis that cis-regulatory variants are the dominant contributors to the AS divergence in mouse strains. And combining these results with previous similar results between human and mouse, and human and chimp, it is possible to say that this hypothesis holds true for mammals in general. · Using F1 hybrid the authors are able to identify unambiguously cis-regulatory divergence allele-specific splicing patterns, since the nascent RNA transcripts form both parental alleles are subject to the same trans-regulatory environments.

· Good statistical analysis - the authors make use of previously defined Bayesian factor thresholds for estimating the statistical significance of the splicing difference.

· Use of two strains divergent over 1.5 MY similar to Drosophila - for comparison purpose.

Points that can use improvement:

1. Why does the percentage of differential splicing events converage of the A3SS is lower (2.8%) compared to the coverage of all the other events (~4.5%)

A: Although the percentage of A3SS divergent events between C57BL/6J and SPRET/EiJ parental strains was significantly lower compared to other splicing events in both fibroblast cell lines and liver tissues, such difference was not observed in the comparison between C57BL/6J and CAST/EiJ strains. It is not clear to us why it is only observed in C57BL/6J *vs* SPRET/EiJ comparison. We feel this is not an important point relevant to our main results and conclusion, and therefore not needed for further discussion. In the revised manuscript, we deleted the sentence and the

column in Table 1 to avoid any misinterpretation.

Minor points

1. In the "Predominant contribution of cis-regulatory variants..." section, they say only 30% of the F1 (C57BL/6J-CAST/EiJ) reads were mapped unambiguously compared to their original F1 hybrid where they were able to map 60 % of the reads, however earlier in the section they say that actually only 72.8 out of 170 million reads were mapped unambiguously, and this actually makes for 40%, (not 60%)

A: Sorry for causing such misunderstanding. Here 40% was calculated by dividing the mapped reads with unambiguous allelic origin (72.8 in previous version) by total number of sequencing reads (170). To calculate the percentage of F1 reads with unambiguous assignment of allelic origin, we should divide the unambiguously mapped reads (72.8) by total number of mappable reads (118.5), *i.e.* 60%. In the revised Table E1, we explained explicitly how we calculated these numbers.

2. Out of the 234 F1 AS events that contain 140 cis- and 38 trans- divergence, what happens with the rest of 56 are they neither cis nor trans?

A: In our revised manuscript, with much higher sequencing depth, after filtering using mock data, out of the remaining 417 parental divergent AS events, 255 and 62 showed *cis*- and *trans*- divergence, respectively. Of these, there are 16 events exhibiting both *cis*- and *trans*- divergence. So there are a total of 116 events not accounted for.

In this study, we required a significant divergent AS events to pass a threshold of BF>5 in all the three replicates and average |ΔPSI|>0.1 (Please see Materials and

methods). Out of the 116 "missing" events, 81 were not identified as *cis*-divergent in the allelic comparison because they did not pass the threshold of BF>5 in all three replicates of F1 data and 35 events with BF>5 in all three replicates, but average $|\Delta PSI| \leq 0.1$. These 116 events neither pass the significant test as *trans*-events.

3. Also since only 219 F1 AS events are above the threshold for being divergent using BF >5 and dPSI > 0.1, what proportion of the 234 events are actually divergent by passing the test?

A: As explained above, out of 417 parental divergent AS events (previously 234), 255 (previously 140) were identified as *cis*- divergent, *i.e.* passing a threshold of BF>5 in all the three replicates and average $|\Delta PSI|>0.1$.

4. The novelty of the study can be questionable since a dominant cis-contribution to the AS in mammals has been observed before in comparative studies in human& mouse (Barbosa-Morais 2012) and human& chimp (Lin 2010), but this study reinforces the generality of the hypothesis over the mouse strain.

A: As elaborated in both Introduction and Discussion of our manuscript, the study of Barbosa-Morais *et al.*, only investigated 13 human/mouse ortholog exons from chromosome 21. The global contributions of *cis*- and *trans*-effects towards divergence of AS in mammals remained unclear. The study by Lin et al. was solely based on the observation of higher sequence divergence flanking divergent AS events and there is no direct measurement of either *cis*- or *trans*- AS divergency. In addition, both studies only investigated exon skipping. To the best of our knowledge, our study is the first direct measurement of global contributions of *cis*- and *trans*-effects towards divergence of all five different major types of AS in mammals.

5. While the discussion of the AS in fly highlights the differences between the two studies it does not give a satisfactory explanation of the dominance of trans-effects in the exon skipping events in fly and merely states the obvious, so I believe that the fly part does not add anything to the conclusion of the paper.

A: We feel it is important to point out the different observations in the relative *cis*-contribution to exon skipping between our study of mouse and previous study of *Drosophila*. We tried our best to provide a likely still non-exhaustive list of possible explanations. We feel such discussion is important for the community to move forward with future studies.

References

Chang, T.H., Huang, H.Y., Hsu, J.B., Weng, S.L., Horng, J.T., and Huang, H.D. (2013). An enhanced computational platform for investigating the roles of regulatory RNA and for identifying functional RNA motifs. BMC Bioinformatics *14 Suppl 2*, S4.

Dodt, M., Roehr, J.T., Ahmed, R., and Dieterich, C. (2012). FLEXBAR-Flexible Barcode and Adapter Processing for Next-Generation Sequencing Platforms. Biology (Basel) *1*, 895-905.

Sterne-Weiler, T., Martinez-Nunez, R.T., Howard, J.M., Cvitovik, I., Katzman, S., Tariq, M.A., Pourmand, N., and Sanford, J.R. (2013). Frac-seq reveals isoform-specific recruitment to polyribosomes. Genome Res *23*, 1615-1623.

Wang, L., Wang, S., and Li, W. (2012). RSeQC: quality control of RNA-seq experiments. Bioinformatics *28*, 2184-2185.

2nd Editorial Decision 20 May 2015

Thank you again for submitting your work to Molecular Systems Biology. We have now heard back from the two referees. As you will see, they are now supportive and we will be able to accept your manuscript for publication pending the following minor points:

As a matter of course, please make sure that you have correctly followed the instructions for authors as given on the submission website.

Thank you for submitting this paper to Molecular Systems Biology.

REFEREE REPORTS

Reviewer #2:

The authors have very convincingly addressed all of our concerns.

Reviewer #3:

The authors answered satisfactorily the reviewers comments. The revised manuscript gives a more detailed presentation of the work.
